# Supplementary material for: Ontology-driven analysis of marine metagenomics: what more can we learn from our data?
Source: Gigascience. 2023 Nov 6;12:giad088. doi: 10.1093/gigascience/giad088 (PMC10632069; doi:10.1093/gigascience/giad088)
Supplement: giad088_GIGA-D-23-00056_Revision_2 [file giad088_giga-d-23-00056_revision_2.pdf]

GigaScience

Ontology-Driven Analysis of Marine Metagenomics: What More Can We Learn from Our Data?

--Manuscript Draft--

Manuscript Number:	GIGA-D-23-00056R2
Full Title:	Ontology-Driven Analysis of Marine Metagenomics: What More Can We Learn from Our Data?
Article Type:	Research
Funding Information:	National Science Foundation (OCE-1639614) — Dr. Bonnie Hurwitz<br>National Science Foundation (CISE-1640775) — Dr. Bonnie Hurwitz<br>Simons Foundation (481471) — Dr. Bonnie Hurwitz<br>Gordon and Betty Moore Foundation (GBMF 8751) — Dr. Bonnie Hurwitz<br>Academy of Finland (339172) — Dr. Alise Ponsero
Abstract:	The proliferation of metagenomic sequencing technologies have enabled novel insights into the functional genomic potentials and taxonomic structure of microbial communities. However, cyberinfrastructure efforts to manage and enable the reproducible analysis of sequence data have not kept pace. Thus, there is increasing recognition of the need to make metagenomic data discoverable within machine searchable frameworks compliant with the FAIR principles for data stewardship. Although a variety of metagenomic web services exist, none currently leverage the hierarchically structured terminology encoded within common life-science ontologies to programmatically discover data. Here, we integrate large-scale marine metagenomic datasets with community-driven life-science ontologies into a novel FAIR web service. This approach enables the retrieval of data discovered by intersecting the knowledge represented within ontologies against the functional genomic potential and taxonomic structure computed from marine sequencing data. Our findings highlight various microbial functional and taxonomic patterns relevant to the ecology of prokaryotes in various aquatic environments. Most importantly, we present and evaluate a novel Semantic Web architecture that can be used to ask novel biological questions of existing metagenomic datasets. Finally, the FAIR ontology searchable data products provided by our API can be leveraged by future research efforts.
Corresponding Author:	Bonnie Hurwitz<br><br>UNITED STATES
Corresponding Author Secondary Information:	
Corresponding Author's Institution:	
Corresponding Author's Secondary Institution:	
First Author:	Kai Blumberg, Ph.D.
First Author Secondary Information:	
Order of Authors:	Kai Blumberg, Ph.D.<br>Matthew Miller<br>Alise Ponsero<br>Bonnie Hurwitz
Order of Authors Secondary Information:	
Response to Reviewers:	We have updated the manuscript with the few minor formatting and editorial requests.

Powered by Editorial Manager® and ProduXion Manager® from Aries Systems Corporation

Additional Information:	
Question	Response
Are you submitting this manuscript to a special series or article collection?	No
**Experimental design and statistics**<br><br>Full details of the experimental design and statistical methods used should be given in the Methods section, as detailed in our Minimum Standards Reporting Checklist. Information essential to interpreting the data presented should be made available in the figure legends.<br><br>Have you included all the information requested in your manuscript?	Yes
**Resources**<br><br>A description of all resources used, including antibodies, cell lines, animals and software tools, with enough information to allow them to be uniquely identified, should be included in the Methods section. Authors are strongly encouraged to cite Research Resource Identifiers (RRIDs) for antibodies, model organisms and tools, where possible.<br><br>Have you included the information requested as detailed in our Minimum Standards Reporting Checklist?	Yes
**Availability of data and materials**<br><br>All datasets and code on which the conclusions of the paper rely must be either included in your submission or deposited in publicly available repositories (where available and ethically appropriate), referencing such data using a unique identifier in the references and in the “Availability of Data and Materials” section of your manuscript.	Yes

Powered by Editorial Manager® and ProduXion Manager® from Aries Systems Corporation

Have you have met the above requirement as detailed in our Minimum Standards Reporting Checklist?	

Powered by Editorial Manager® and ProduXion Manager® from Aries Systems Corporation

Manuscript

Click here to access/download;Manuscript;Blumberg_et_al_2023_for_producti

Ontology-Driven Analysis of Marine Metagenomics: What More Can We Learn from Our Data?

Kai Blumberg[1,2] kblumberg@arizona.edu 0000-0002-3410-4655

Matthew Miller[2] mattmiller899@arizona.edu 0000-0002-3491-8763

Alise Ponsero[1,2,3] alise.ponsero@helsinki.fi 0000-0002-4125-7561

Bonnie Hurwitz[1,2] bhurwitz@email.arizona.edu 0000-0001-8699-957X corresponding author

[1]Department of Biosystems Engineering, University of Arizona, Tucson, Arizona

[2]BIO5 Institute, University of Arizona, Tucson, Arizona

[3]Human Microbiome Research Program, Faculty of Medicine, University of Helsinki, Helsinki, Finland

Abstract

Background

The proliferation of metagenomic sequencing technologies have enabled novel insights into the functional genomic potentials and taxonomic structure of microbial communities. However, cyberinfrastructure efforts to manage and enable the reproducible analysis of sequence data have not kept pace. Thus, there is increasing recognition of the need to make metagenomic data discoverable within machine searchable frameworks compliant with the FAIR principles for data stewardship. Although a variety of metagenomic web services exist, none currently leverage the hierarchically structured terminology encoded within common life-science ontologies to programmatically discover data.

Results

Here, we integrate large-scale marine metagenomic datasets with community-driven life-science ontologies into a novel FAIR web service. This approach enables the retrieval of data discovered by intersecting the knowledge represented within ontologies against the functional genomic potential and taxonomic structure computed from marine sequencing data. Our findings highlight various microbial functional and taxonomic patterns relevant to the ecology of prokaryotes in various aquatic environments.

Conclusions

In this work, we present and evaluate a novel Semantic Web architecture that can be used to ask novel biological questions of existing marine metagenomic datasets. Finally, the FAIR ontology searchable data products provided by our API can be leveraged by future research efforts.

Keywords

Ontology, Metagenomics, Microbial Ecology, RDF, FAIR Data

Background

Elucidating the complex ecological relationships between the taxonomic structure, the functional genomic potentials of microbial communities and environmental factors has long been an important focus of microbial ecology. With the advances of Whole Genome Sequencing (WGS) technologies an unprecedented quantity of sequencing data has been collected from numerous environments [1–3]. This is especially true of marine environments, where microbes have been shown to play critical roles in maintaining food webs [4], driving biogeochemical cycling of elements [5], and regulating climatic conditions [6]. Regarding data management there has been much discussion of the FAIR principles (Findability, Accessibility, Interoperability, and Reusability), which outline general principles for improving the digital ecosystem supporting the publication of scientific data [7]. Over the years, several web-portals, tools and databases have been developed for the management and analysis of metagenomic data including the metagenomics RAST server [8], the Quantitative Insights Into Microbial Ecology (QIIME) [9], the microbiome analysis resource MGnify [10], and the Genomes OnLine Database (GOLD) [11]. Additionally, the Minimum Information about any (x) Sequence (MIxS) checklists were developed to help standardize metadata accompanying the sequencing data to allow for its reuse or meta-analyses [12]. Despite the existence of analysis tools and data reporting standards, the proliferation of sequencing data has outpaced the efforts of existing cyberinfrastructure systems to collect, process and analyze sequence data in an automated and reproducible manner. Recent Initiatives such as the National Microbiome Data Collaborative (NMDC) strive to reduce these barriers by providing infrastructure, tooling, and technologies to support reproducible and cross-study analyses of sequencing data [13]. A chief concern of the NMDC and related initiatives is to foster a digital knowledge ecosystem for sequencing data that

is consistent with the FAIR guiding principles for data management. The authors of the FAIR principles present a vision in which data is made discoverable to machine agents deployed in programmatic search routines over data annotated with common vocabularies and represented in machine readable frameworks [7]. In the FAIR publication, the authors suggest the use of Semantic Web technologies such as the Resource Description Framework (RDF) to serve as a machine-readable data and knowledge representation framework, and web-accessible ontologies — hierarchically-structured informatic systems for knowledge representation — to serve as vocabularies for data annotation. Due to their machine-processable linkages between represented entities, ontologies are recommended above other types of controlled vocabularies to make data FAIR. A longstanding coordinated ontology development effort predating the FAIR principles is the Open Biomedical and Biological Ontologies (OBO) Foundry and Library [14]. OBO foundry ontologies represent terminology from a large variety of life science domains, and together serve as a unified and interoperable multidisciplinary knowledge representation model [15]. The OBO foundry includes the Gene Ontology (GO), which provides representations of the biological processes and molecular functions of genes [16] and is widely used for the annotation of genomic sequencing data [17]. Other OBO ontologies include the Environment Ontology (ENVO) for environment types and environmental parameters [18,19], as well as NCBITaxon, the ontology representation of the National Center for Biotechnological Information (NCBI) organismal taxonomy database [20].

However, despite the importance placed on using ontologies to make metagenomic data FAIR, web-based resources that enable the use of terminology from ontologies to assist in programmatically searching for the functional, and taxonomic contents of metagenomic data are still lacking. To meet this challenge with marine microbiome data, we previously developed a web resource, Planet Microbe, that uses ontologies to make data from numerous state-of-the-art marine metagenomic studies programmatically searchable by their environmental and

physicochemical contextual data [21,22]. In terms of tools for computing and annotating functional genomic information from metagenomic data, there exist a variety of resources such as the Clusters of Orthologous Groups of proteins (COGs) database, [23], the Kyoto Encyclopedia of Genes and Genomes (KEGG) database [24], and the SEED genome annotation database [25]. Unlike these resources that use flat controlled vocabularies for annotation, GO is an ontology with a hierarchical structure that allows for the programmatic discovery of sub concepts within its terminology hierarchies. Resources such as MGnify provide GO annotation frequency tables computed from metagenomic samples, however, those results are currently not programmatically searchable in a way that leverages the hierarchical structure of GO to discover data. Although the original Planet Microbe web portal made environmental contextual data programmatically searchable using terminology from OBO ontologies, it currently does not support this functionality for functional genomic and taxonomic data.

To fill this gap, we built upon the previously published Planet Microbe web portal to propose a novel FAIR architecture for the ontology-driven harmonization and meta-analysis of large-scale marine metagenomic datasets. We demonstrate how this type of effort can enable the discovery and retrieval of data about the environmental context, functional genomic potential, and taxonomic structure of the marine prokaryotic microbiome through an API service. The system consists of a new RDF database containing data annotated with terminology from OBO Foundry ontologies including GO, ENVO and NCBITaxon. The structure of the database allows for data to be searchable using the hierarchical structure of ontologies, allowing for the discovery of information relevant to specific biological questions. The database created in this work is publicly available via an API, along with customizable scripts to query and analyze new questions of interest to future researchers. The documentation is available from the following link [26]. Here, we present, validate, and analyze results derived from our new FAIR marine metagenomic data discovery framework. We anticipate the Semantic Web architecture

presented here will aid future researchers to discover data by which to further examine their own hypotheses about the structure and function of microbial communities in the oceans. Moreover, the FAIR data products that are queryable through our API constitute a microservice that can be leveraged by future projects connecting to other data sources.

MATERIAL AND METHODS

Marine metagenomic data selection

We built upon the Planet Microbe database (RRID:SCR_024478) [21], available online from [27], using it as the source of marine metagenomic data for this work. First, we selected prokaryote enriched metagenomes from the database by using the website's search interface to get samples based on minimum and maximum filter sizes ranging from 0.2 to 3 micrometers. We further constrained the results by the NCBI metadata "Strategy" field taking only samples of type Whole Genome Sequencing and Whole Genome Analysis. Next, the richness of GO functional and NCBITaxon species annotations, (derived from the pipeline described in the next section) against the number of reads was plotted and used to identify relevant sequencing depth cut-offs (Supplementary Figure 1). Analyzing the richness curve shown in Supplementary Figure 1 in a manner analogous to a rarefaction curve, the number of new functional and taxonomic annotations seen with increasing sample sizes reaches a plateau between 5 and 10 million reads. Hence metagenomes with less than 5 million reads were discarded, and the remaining metagenomes were subsampled to 10 million reads prior to taxonomic and functional annotation. After annotation, low quality samples were removed by checking the NCBITaxon annotation richness against the number of Open Reading Frames (ORFs). A minimum threshold of 10,000 unique NCBITaxon annotations per sample was chosen. (Supplementary Figure 2). All in all, a final set of 819 samples remained for the integration into the new system.

Functional and Taxonomic Metagenomic Annotation

The High Performance Computing (HPC) Simple Linux Utility for Resource Management (SLURM) based pipeline was used for the Functional and Taxonomic annotation of metagenomic data, and is available from the following Github repository [28]. Briefly, the pipeline consists of three main steps for 1) the quality control of raw metagenomic reads, 2) their taxonomic annotation, and 3) their functional annotation.

The steps of the quality control pipeline are as follows. First, the alignment algorithm bowtie2 (v2.4.2) was used to remove reads mapping to Phix and human genomes that are presumed to be contamination or artifacts from sequencing [29]. Next, Trimmomatic (v0.39) was used to trim adaptor sequences from reads [30]. Finally, vsearch (v2.21.1) was used to quality control fastq sequences by removing low quality reads [31].

The Taxonomic annotation pipeline consisted of taking the reads that passed the quality control pipeline and running them through the k-mer-based taxonomic classification software Kraken2 [32] using the PlusPF database versioned from 01/27/2021, which is available from [33]. This produced taxonomic count tables annotated with NCBITaxon identifiers.

The Functional annotation step of the pipeline is based on the European Bioinformatics Institute (EBI) microbiome analysis resource's [10] Pipeline 4.1, available from [34]. After quality control, FragGeneScan (v1.31) was run to predict gene ORFs [35]. FragGeneScan in turn made use of the Prokaryotic Dynamic Programming Genefinding Algorithm (Prodigal) (v2.6.3) for the prediction of bacterial and archaeal protein-coding genes [36]. Next, InterProScan (v5.46-81.0) [37,38] was run to annotate the predicted protein-coding genes with InterPro protein annotations, as well as mappings to GO classes. InterPro matches were generated against

predicted coding sequence regions using only the Pfam database (v33.1) [39]. Finally, the parallelized InterPro and GO annotation files were merged into singular final functional annotation TSV files.

Finally, the results of the functional annotation pipeline run on all samples, as well as metadata about the job statistics derived from the functional and taxonomic annotation pipeline, such as the computed sample numbers of ORFs and reads were uploaded to the Zenodo data repository. The dataset is available from [40].

Semantic Web Data Integration Pipeline

RDF Database Construction

The RDF formatted versions of both the functional and taxonomic count data, as well as the environmental, physicochemical, and spatiotemporal contextual data associated with each sample were loaded into an Apache Jena TBD2 RDF database (v4.3.2) [41]. The Planet Microbe Ontology, available from [42], which contains a subset of the classes from the ENVO, was loaded into the RDF database. Additionally, we created subsets of the GO and NCBITaxon ontologies that contained only the set of ontology classes present in the functional and taxonomic sample annotation data, along with their recursive parent classes. These subsets were created using the ROBOT (v1.8.3) command line tool's extract module [43] and were also added into the RDF database. Finally, the database was loaded into an instance of an Apache Jena Fuseki2 (v4.3.2) SPARQL server. Scripts for the creation and use of the new RDF database are available from the following repository [44].

Computed Functional and Taxonomic RDF Data Integration:

Outputs from the functional and taxonomic analysis pipeline were parsed and merged into final count tables using custom python3 scripts (see Data Availability). These tables including GO and NCBI Taxon identifiers and their accompanying count values were converted into RDF and loaded into the RDF database using the Tarql (v1.2) command line software. The SPARQL construct queries used to build the RDF graph database can be found in the “triplestore” subdirectory of the Planet Microbe Semantic Web Analysis Github repository [44]. Examples illustrating the RDF graph structure used in this work can be found in the Appendix 2 of the protocol shown in the Data Availability section. Additionally, metadata about the job statistics derived from the functional and taxonomic annotation pipeline, such as the computed sample numbers of ORFs and reads were also converted into RDF format and loaded into the RDF database.

Environmental, Physicochemical and Spatiotemporal Data Integration:

Physicochemical and spatiotemporal metadata attributes (e.g., temperature, latitude, and environmental medium) were queried from the API of the Planet Microbe database [21,27], which is available from [45]. These contextual environmental variables were previously harmonized and curated as described in [21,22]. JSON data retrieved from API calls to the Planet Microbe database, see README file from [46], were parsed into TSV using custom python3 scripts, and then subsequently converted to RDF format and loaded into the new RDF database using Tarql and additional custom SPARQL construct queries.

Database SPARQL Queries and Data Processing

The RDF database hosted as a public web service can be accessed using SPARQL queries generated from a custom python3 script, see Data Availability.

Statistical Analyses

Data Preprocessing

All statistical analyses and data visualizations were conducted in R (v4.2.2). The data used was preprocessed as follows. The functional and taxonomic results shown in all figures except for Table 1, were normalized as follows. Functional annotation count values were normalized by dividing each sample's GO count values by the number of ORFs annotated by the pipeline for each sample. Taxonomic annotation count values were normalized by dividing each sample's NCBITaxon count values by the number of reads that remained after the taxonomic pipeline's quality control steps for each sample. In contrast the results in Table 1 were based on the raw (un-normalized) gene count data. The genus level depth profiles shown in Figure 1A included the summation of the relativized counts of all taxonomic levels including and below the genus level for the given groups. The depth profiles in Figure 3 only show the relative abundance of only one species per plot. For the regression analyses using taxonomic data, additional preprocessing steps included 1) filtering to keep only species and strain level results, as well as 2) a prevalence filter in which taxonomic groups that were present in less than 1 count per million in 5% of samples were removed as potential contaminants. Finally, in both functional and taxonomic regression analysis, the data was normalized via Aitchison (centered log-ratio) transformation to account for compositionality of sequencing data [47]. Aitchison transformations were performed using the "compositions" R package (v2.0-5).

Statistical Tests and Data Visualization

Data products retrieved from the RDF database were analyzed using custom R scripts for data visualization, statistical analysis, and machine learning methods (see Data Availability). All figures were generated using the R "ggplot2" package (v3.4.0). All Spearman correlations were performed in R using the "cor" package (v4.2.2). Permanova tests were performed using the

adonis2 function with Euclidean distances and 999 permutations from the R “vegan” package (v2.6.4). Permutation tests for homogeneity of multivariate dispersions were performed using the vegan betadisper function with Euclidean distances on distance matrices using Aitchison transformed data created with a Euclidean distance.

Elastic Net Linear Regression Analysis

Elastic net linear regression analyses were performed using the R “glmnet” package (v4.1.6) [48]. We chose to do linear regressions using the Elastic net method as it incorporates features from both the lasso and ridge regression methods, each of which are popular methods for linear regression [48]. We performed the linear regression analyses using the default glmnet parameters including the use of 10-fold cross-validation, as well as the selection of a regularization parameter lambda such that the cross-validated error is within one standard error of the minimum in order to determine the model coefficients. We additionally excluded the 30% of the data with the greatest variance from the analysis as per the recommendation for genomic data [48]. The analyses regressing genes against physicochemical parameters shown in Figure 2 and Figure 4, as well as regressing species against other species to determine the species shown in Figure 3 used a Gaussian family for the objective function. The analyses regressing genes or species against binned river or marine environment types used a Binomial family for the objective function.

Indicator Species Analysis

Indicator species analysis on gene families differentiating oxic and anoxic samples, shown in Table 1, was conducted using the multipatt function from the R “indicspecies” package (v1.7.12) with 999 permutations.

System Overview Figure

A graphic overview representation of the materials and methods workflow described in this manuscript is shown in Figure 5, which includes a summary of all web links to repositories, databases and protocols in the figure caption.

RESULTS AND DISCUSSION

System Overview

In this work, we describe a novel cyberinfrastructure system using Semantic Web technologies for the analysis and integration of functional and taxonomic and physicochemical data. Our system consists of an RDF triplestore database, which we populated with the outputs of a functional and taxonomic annotation pipeline run on the prokaryotic subset of marine metagenomic samples from the Planet Microbe Database [21,22]. We also populated the new RDF database with a subset of accompanying MIxS compliant metadata about the environmental context and measured physicochemical parameters, sourced from the upstream database. The final components added to the RDF database are interoperable life-science ontologies from the OBO foundry including the GO, NCBITaxon and the application ontology for the Planet Microbe Database, the Planet Microbe Ontology (PMO), which includes terminology from the ENVO. Being a Semantic Web database, our RDF triplestore uses terminology represented as Ontology Web Language (OWL) classes that are sourced from the above ontologies to pair data with machine-readable semantic descriptions of such data. Specifically, the outputs of the functional and taxonomic genomic annotation pipeline are gene and species counts labeled with classes from the Gene Ontology, and NCBITaxon respectively. Additionally, the MIxS compliant environmental contextual data as well as physicochemical parameters are annotated with classes from ENVO and PMO.

Within ontologies, classes are formally encoded with ancestor/descendant relationships to other classes, giving ontologies a hierarchical graph structure. This structure can be leveraged to recursively search an ontology for subclasses of a given class, enabling the discovery of new information. For example, searching GO for recursive subclasses of '*cellular metabolic process*' (GO:0044237) yields '*photosynthesis*' (GO:0015979) as well as '*methanogenesis*' (GO:0015948) as both are descendent classes that are types of '*cellular metabolic process*' (GO:0044237). These searches can be performed by humans visualizing ontologies, as well as by automated machine search routines. Our RDF system can not only leverage this machine search capability to find relevant classes within ontologies, but it can also find the intersection of the ontology-discovered classes with any data annotated with such classes. By combining life-science ontologies with ontology-annotated data in a machine searchable RDF system, we enable a novel ontology-driven genomic feature selection technique. This enables expert information about species taxonomy and gene functions encoded within ontologies to be used to automatically discover genomic information relevant to a question of interest.

Because ontology classes also contain human readable labels, we can perform this feature selection method by posing natural language questions in which subjects or objects of a natural language question are ontology class labels. For example: "What data do we have about any '*cellular metabolic process*' (GO:0044237) occurring in samples sourced from the '*sea surface layer*' (ENVO:01001581)?". Our system's query script can be leveraged by other users to discover data by which to answer such natural language questions by specifying classes from the relevant ontologies as input arguments. This is done within the query script by assembling a SPARQL query based on the classes given as input arguments and then posting the SPARQL query to systems SPARQL endpoint. Finally, data resulting from query is returned, which in turn can be analyzed using statistical and or machine learning workflows. It should be noted that

performing machine searches on data annotated with interoperable vocabularies (such as ontology classes), which are stored within computable representational frameworks (such as RDF) is the intention behind the FAIR data principles [7].

System Validation

To evaluate if our novel system produced biologically expected results, we performed a series of queries for species, genes, and physicochemical parameters with known spatial distributions using the HOT 224-283 dataset [49] sampled from the very well-studied Hawaiian Ocean Time (HOT) series study's Aloha station [50,51,49]. We specifically chose this state-of-the-art data set from a well-studied location in order to validate the capacity of the system to produce expected taxonomic, functional genomic and physicochemical results.

We began our validation process by seeing if the system can recapitulate known trends about species taxonomy. The HOT 224-283 dataset includes cell counts, for a subset of samples with measured values for the abundance of cells from both the *Prochlorococcus* and *Synechococcus* genera. Both genera are well studied photosynthetic members of the Cyanobacteria phyla known to be in high abundance at the HOT Aloha station [49,51]. The *Prochlorococcus* genus is known to be one of the most dominant phytoplankton species in the tropics and subtropics, which can account for up to 43% of the photosynthetic biomass in oligotrophic conditions, with a depth range down to 200 meters [52]. Together, *Prochlorococcus* and *Synechococcus* contribute about 25% of primary production in the oceans, making them among the most abundant photosynthetic organisms on Earth [53,54]. Hence, we selected these genera to test the genomically-derived taxonomic results produced by our system against the cell count data measuring the same phenomena using a different method. Although the methods are not directly comparable as the cell counts are concentrations and the genome-derived results are

compositions, we quantified the relationship between the two measures of the phenomena using Spearman correlations.

To make this comparison, we used our system to query for all samples from the HOT 224-283 dataset with taxonomic assignments matching sub lineages of both the ‘*Prochlorococcus*’ (NCBITaxon:1218) and ‘*Synechococcus*’ (NCBITaxon:1129) genera. We additionally queried the system for ‘depth of water’ (ENVO:3100031) values, as well as for *Prochlorococcus* and *Synechococcus* cell counts, (PMO:00000159) and (PMO:00000160) respectively. In order to report on the *Prochlorococcus* and *Synechococcus* taxonomic annotations at the genera level, we took the summation of the relative abundance of all taxonomic counts assigned within each lineage. The results are plotted as depth profiles in Figure 1A. Comparing read-based taxonomic assignments of *Prochlorococcus* and *Synechococcus* reads against corresponding cell count measurements we observed Spearman correlation values of 0.601 and 0.826 respectively. The high positive correlation value observed between *Synechococcus* reads and cell counts is encouraging as a sanity check. The moderate strength correlation between the sum of *Prochlorococcus* reads and cell counts is most likely due to the presence of low light adapted *Prochlorococcus* strains, previously reported at HOT station Aloha [22,55], which follow a different depth distribution. Hence the low light-adapted strains may be causing the difference in signal between two measurements of the same phenomena, see Figure 2 and the Associations Between Species section for further details.

It is important to note that the purpose of the system presented here is to harmonize and integrate metagenomic data within its ecological context. As such an even more relevant test of the system — rather than comparing methods used to get taxonomic information against other methods — is to test if the taxonomic results derived from using the system are ecologically meaningful. Returning to the *Prochlorococcus* and *Synechococcus* genera that are phototrophs

known to be abundant at the surface of this ecological context, it is expected that the relative abundance of these organisms should decrease with depth due to decreasing light availability. Thus, we also tested the correlations of the derived taxonomic results against depth. As expected, we observed strong anti-correlations between the sum of relativized *Prochlorococcus* and *Synechococcus* reads with depth, with high Spearman correlation values of -0.851, and -0.811 respectively.

After confirming that the system can recapitulate expected trends regarding the depth distributions of abundant photosynthetic organisms; we used the metabolic process of photosynthesis to test if the system can also produce ecologically meaningful results about the functional genomic capacity of ecosystems under study. To explore this, we used the system to retrieve samples from the HOT 224-283 dataset with any functional genomic annotation count values corresponding to '*photosynthesis*' (GO:0015979) from the Gene Ontology (GO) and plotted their relative abundances against depth in Figure 1B. As expected, the relative abundance of photosynthesis genes, which is limited by light availability [56,57], is highest at the surface, decreasing with depth. Quantifying the relationship between photosynthesis gene relative abundance and depth with a Spearman correlation, we found a high negative correlation value of -0.873.

Another mode of energy generation known to occur in very different biogeographic distributions to photosynthesis is methanogenesis, the conversion of organic matter to methane [56,58]. Methanogenesis is limited by oxygen inhibition and typically constrained by depth and oxygen profiles [59,60,58]. In marine systems such as the HOT aloha station, methanogenesis is expected to increase with increased depth and concomitant decreased oxygen concentrations. We used the system to query for all HOT 224-283 samples with '*methanogenesis*' (GO:0015948) annotations, as well as depth. The results of which are shown in Figure 1B. As

expected, our derived results showed that methanogenesis increased with depth, with a high Spearman correlation value of 0.845.

It should be stressed that another important feature of this data harmonization and retrieval system is the ability to retrieve the functional or taxonomic data within its ecological context, especially in relation to physicochemical gradients. In addition to depth, the example HOT 224-283 dataset includes several other measured parameters including oxygen and phosphate concentrations which are also searchable in the system via their ontology annotations. As oxygen gradients are also important in shaping the biogeography of methanogenic processes [58], we used the system to additionally query for samples with measured values for '*concentration of dioxygen in liquid water*' (ENVO:3100011). Using the discovered intersection of the oxygen and methanogenesis gene results, we were able to quantify the relationship between them. As expected, we found a strong inverse correlation between these variables, with a Spearman correlation value of -0.837.

Finally, we tested one more example of a known relationship between functional genomic capacities and physicochemical gradients regarding phosphorus, an element essential for the growth of all organisms [61,62]. In marine systems, especially at the surface, phosphorus availability can limit growth, as well as affect the taxonomic structure of microbial communities [63–66]. In the ocean, phosphorus is bioavailable in the form of dissolved inorganic phosphate [62] and follows a nutrient-type depth profile with low concentrations at the surface and an increase with depth [67]. This phosphate distribution is well known to occur in the Pacific Subtropical Gyre, specifically at the HOT station Aloha [68]. Thus to ensure that our system is able to recapitulate information on this well-known phenomena, we used it to query for samples from the HOT 224-283 dataset with both '*phosphate ion binding*' (GO:0042301) genes as well as measured values for 'concentration of phosphate in liquid water' (ENVO:3100026), see

Figure 1C. Examining the relationship between the relative abundance of phosphate ion binding genes and measured phosphate concentration, we observed a strong inverse Spearman correlation of -0.802. As expected, we found an inverse relationship between phosphate binding genes and measured phosphate concentration as cells limited by available phosphate will be in greater need of phosphate binding genes to be able to uptake it. This can clearly be seen in Figure 1C where at the surface phosphate concentrations are low and increasing with depth, while phosphate ion binding gene abundance shows the opposite trend. These and the preceding results help to sanity check that the data integration system is capable of producing meaningful results when comparing known biogeographic patterns.

Associations of Genes and Physicochemical Factors

Moving on from known ecological questions, we next explore the capacity of the system to discover and address new or ongoing questions, the answers to which might not be fully established. The following sections provide case studies of using this FAIR data metagenomic data integration system to discover data relevant to specific ecological questions. The data discovery and analysis workflows presented here could serve as a blueprint for further investigations of new questions concerning marine microbiology using the Planet Microbe SPARQL endpoint, or future cyberinfrastructure systems specific to other scientific domains of interest.

As a first example of using the system to ask and answer novel questions of interest, we choose a question exploring the associations between a gene family of interest and a physicochemical factor. Specifically, we asked the question: "What '*cation binding*' (GO:0043169) genes are most associated with shallow, intermediate, and deep 'water depth' (ENVO:3100031) ranges in samples from the HOT Aloha 224-283 dataset?".

To provide answers to this question, we employed the following workflow. First, we queried the system's SPARQL endpoint to retrieve the counts values of genes annotated with GO terms from the ‘*cation binding*’ (GO:0043169) hierarchy. This is achieved by performing a recursive subclass query to identify all the terms that are within the GO class hierarchy of interest and then retrieve all samples that have count values corresponding to functional genomic annotations with such terms. The query was further refined to only include samples from the HOT 224-283 dataset that also had measured values for ‘*water depth*’ (ENVO:3100031). Next, we analyzed the data with an elastic net linear regression model with a gaussian distribution, to perform additional feature selection to identify which of the discovered genes change most with depth. In the regression analysis, we used the GO class counts as the predictor variables, and depth as the response variable.

The final elastic net linear regression model reduced the number of genes from the original 17 unique GO gene types discovered in the query down to five, which are plotted with bars binned by depth into shallow, intermediate, and deep depth ranges in Figure 2. In order to test the overall significance of the chosen depth bins on all 317 genes discovered in the original query, we performed a Permanova test with 999 permutations which showed the depth bins to be significant with a p-value of 0.001. We additionally performed a permutation test for homogeneity of multivariate dispersions which showed non-significant variance with a P value of 0.255. The regression analysis showed that ‘*calcium ion binding*’ (GO:0005509) was the most abundant cation binding gene which decreased in abundance with depth. The increase in calcium ion binding genes at the surface is most likely due to the fact that Cyanobacteria, which are abundant in HOT Aloha surface samples, are known to be an important driver of calcium carbonate precipitation by producing extracellular polysaccharides, which act as binding sites for calcium [69,70].

Interestingly, three of the five results were ion binding genes to transition metals. Indeed, previous studies have shown that transition metals play important roles in marine biogeochemical processes including photosynthesis and its accompanying metabolic processes [71]. The first of which, '*nickel cation binding*' (GO:0016151) decreased in abundance with depth. Nickel is a bioactive transition metal, which typically displays a nutrient-type profile within marine systems. Nickel is typically depleted in the photic zone and at higher concentrations at depth, which indicate biological use at the surface and release back into the water column at depth [72,73]. These distribution patterns of nickel in marine ecosystems described in the literature are consistent with the nickel cation binding gene distribution that we observed. The other transition metal ion binding genes discovered in our analysis were '*ferric iron binding*' (GO:0008199) and '*ferrous iron binding*' (GO:0008198). The former decreased in abundance with depth while the latter followed the opposite trend of increasing with depth. Dissolved iron, which typically occurs in seawater at low concentrations in either the ferric (+3) or ferrous (+2) oxidation states, are thought to be the most bioavailable forms of iron [74]. The extent to which ferrous iron is used by microorganisms, however, is not well known [75]. Ferrous iron is mainly produced via photochemical reactions and is usually oxidized quickly back to ferric iron in the presence of oxygen [76], however, ferrous iron can persist in environments with lower temperature and oxygen [77,78]. At HOT station Aloha, trace amounts of iron have previously been measured [79] and using our system to additionally query for oxygen concentration, shown in Supplementary Figure 3, we observe a decrease in oxygen with depth. Taken together these observations explain the increase in ferric iron binding genes at the more oxygenated surface and increase of ferrous iron binding genes with depth where there are lower oxygen values.

The final association identified in this analysis was '*arginine binding*' (GO:0034618), which had the lowest relative abundance of the genes identified in the analysis, and which increased in relative abundance with depth. Arginine is an amino acid used in the biosynthesis of proteins.

Arginine uptake has been shown to co-occur with ammonia uptake as a nitrogen source in the marine diatom *Phaeodactylum tricomutum* [80], however, its uptake by marine microorganisms at depth has not been as extensively studied. Archaeal communities have been shown to take up a variety of other amino acids at both midrange depths (200 meters) of the Mediterranean Sea and Pacific Ocean [81] and in deep mesopelagic and bathypelagic waters of the North Atlantic [82]. Depth has also been shown to be a factor affecting the amino acid uptake of *Prochlorococcus* in the Southern Atlantic tropical gyre [83]. These previous observations fit with our observations here of increased proportions of arginine binding genes with depth, however, this is an example of a potentially novel association discovered using the system that might be worth investigating in subsequent studies.

Associations Between Species

Next, we illustrate an example of using the system to ask and answer questions about associations between species. Again, using the HOT 224-283 dataset we asked the question: “What ‘*Cyanobacteria*’ (NCBITaxon:1117) species have depth distributions most resembling that of the known low light adapted strain ‘*Prochlorococcus sp. MIT 0801*’ (NCBITaxon:1501269) and thus might also be low light adapted?”.

To find answers to this question, we used the system to query for all samples from the HOT 224-283 dataset with any type of ‘*Cyanobacteria*’ (NCBITaxon:1117). Next, we employed an elastic net linear regression model with a gaussian distribution, which used the known low light-adapted strain ‘*Prochlorococcus sp. MIT 0801*’ (NCBITaxon:1501269) [55,84] as the response variable, and the other ‘*Cyanobacteria*’ (NCBITaxon:1117) species as predictor variables. Of the 186 unique Cyanobacteria species, four were selected as final coefficients in the elastic net regression model. Depth plots corresponding to the target species as well as the species identified as coefficients in the regression analysis are shown in Figure 3. We specifically chose

this question as it could be verified by examining the depth profiles of the discovered species to see if they are similarly distributed to the target species that is known to be low light-adapted. All four discovered candidate low light-adapted Cyanobacteria species are of the *Prochlorococcus* genus. Upon examination, the depth profiles of the discovered species, (shown in Figure 2 B-E) reveal their distributions to be much more similar to the distribution of the target species, (shown in Figure 2) than to the distribution of the sum of all *Prochlorococcus* species shown in Figure 1A.

Regarding the first two discovered *Prochlorococcus* isolates '*Prochlorococcus marinus str. NATL1A*' (NCBITaxon:167555) and '*Prochlorococcus marinus str. NATL2A*' (NCBITaxon:59920), it is known that both are low-light adapted, and are referred to as the LLI *Prochlorococcus* clade, or the eNATL2A ecotype [85,86]. The third discovered species '*Prochlorococcus marinus str. MIT 9211*' (NCBITaxon:93059) has also previously been identified as being low light adapted [84]. Finally, the last discovered species '*Prochlorococcus marinus subsp. marinus str. CCMP1375*' (NCBITaxon:167539), previously called Prochlorococcus *marinus SS120*, is also known to be low light adapted [87].

Although none of the results derived from this example are biologically novel, it is encouraging that all the species generated by the system and subsequent analysis as hypotheses to this question have previously been experimentally validated. This demonstrates that the system enables these types of investigations as it can generate correct hypotheses to biological questions. In this and the previous section we demonstrated examples of using the system to find associations between genes or species and physicochemical properties, as well as associations between species. These workflows could also be used to hypothesize associations between genes, as well as between species and genes. It should of course be noted that any

novel findings generated from the system constitute hypotheses which would need to be experimentally validated.

Discovery of Specific Environments and Physicochemical Gradients

Another strength of the proposed system is the ability to search for samples collected from specific types of environments. Indeed, the system integrates MIxS-compliant environmental contextual information, consisting of annotations with classes from ENVO. These annotations, specifying types of environments can additionally be leveraged within our system's search queries, in combination with physicochemical parameters to discover data collected from specific ecosystems that exist within a particular set of conditions.

Consider the following example. Dissolved oxygen concentration is an important physicochemical parameter known to profoundly affect the structure of marine microbial communities [88,89]. In the absence of oxygen, alternative terminal electron acceptors are used for respiration by microbial communities [90]. Marine regions with longstanding low oxygen concentrations, referred to as oxygen minimum zones (OMZs) [91] are of particular interest as they are known to be expanding [90,92] and may even threaten some fisheries [93]. OMZs typically occur between depths of 200 to 1,500 meters in waters below the photic zone where large amounts of sinking organic matter from phototrophs is remineralized without sufficient physical resupply of oxygen [94].

In order to study microbial species associated with OMZs, we formulate the following question: "In '*marine mesopelagic zone*' (ENVO:00000213) samples from a '*depth of water*' (ENVO:3100031) ranging between 300 to 600 meters, what species from a variety of OMZ affiliated phyla — including '*Alphaproteobacteria*' (NCBITaxon:28211), '*Bacteroidetes*' (NCBITaxon:976), '*Cyanobacteria*' (NCBITaxon:1117), '*Deltaproteobacteria*'

(NCBITaxon:28221), '*Epsilonproteobacteria*' (NCBITaxon:29547), '*Firmicutes*' (NCBITaxon:1239), '*Gammaproteobacteria*' (NCBITaxon:1236) and '*Planctomycetes*' (NCBITaxon:203682) — increase in abundance as the '*concentration of dioxygen in liquid water*' (ENVO:3100011) decreases in Tara Ocean samples?".

To answer this question, we queried the system separately for each of the phyla of interest chosen due to having members previously reported as being OMZ associated [89,95–98]. We further constrained each query using the relevant ENVO classes '*marine mesopelagic zone*' (ENVO:00000213), '*concentration of dioxygen in liquid water*' (ENVO:3100011), and '*depth of water*' (ENVO:3100031). The former being defined as the zone immediately below the photic zone, where OMZs are known to occur. Finally, we additionally constrained the query to only search for samples from the Tara Oceans, the project with the largest geographic scope integrated within our systems database. The results of each query were then analyzed using elastic net linear regression models with gaussian distributions where the species from each phylum were used as the predictor variables, and oxygen concentration was used as the response variable (Figure 4).

Several of the identified species are known anaerobes or facultative anaerobes. Within the Alphaproteobacteria phyla, '*Terasakiella sp. SH-1*' (NCBITaxon:2560057), was isolated under microaerophilic conditions [99], and its genome contains genes that could be used to support alternative forms of respiration [100]. '*Methylorubrum extorquens CM4*' (NCBITaxon:440085) is a known anaerobic soil bacterium isolated from a petrochemical factory [101]. The Bacteroidetes strain '*Paludibacter propionicigenes WB4*' (NCBITaxon:694427) is a strict anaerobe, isolated from rice plant residue in anoxic rice-field soil [102]. The Deltaproteobacteria strain '*Maridesulfovibrio salexigens DSM 2638*' (NCBITaxon:526222) is a mesophilic anaerobe, isolated from mud [103]. Within the Epsilonproteobacteria phyla the Black Sea isolate

‘*Candidatus Sulfurimonas marisnigri*’ (NCBITaxon:2740405) respires anaerobically by oxidizing sulfide with manganese (IV) oxide [104]. Additionally, ‘*Sulfurospirillum deleyianum DSM 6946*’ (NCBITaxon:525898), isolated from freshwater pond sediment is microaerophilic, respiring via sulfur reduction coupled to nitrate oxidation [105]. From the Firmicutes phylum, the species ‘*Tetragenococcus osmophilus*’ (NCBITaxon:526944) comes from a genus known to be facultatively aerobic [106]. From the Gammaproteobacteria phyla, members were identified from the Aeromonas genus, which includes facultative anaerobes and are known to be ubiquitous in fresh and brackish water [107]. Finally, the Planctomycetes species ‘*Anaerohalosphaera lusitana*’ (NCBITaxon:1936003) is a known anaerobe isolated from anoxic hypersaline sediments of evaporation ponds [108].

To further explore the effects of oxygen in shaping mesopelagic zones, we used the system to investigate the functional genomic capacities of low oxygen environments. We asked: “What ‘*binding*’ (GO:0005488), ‘*cellular metabolic process*’ (GO:0044237), and ‘*oxidoreductase activity*’ (GO:0016491) genes are indicators of anoxic environments in Tara Oceans ‘*marine mesopelagic zone*’ (ENVO:00000213) samples from 300 to 600 meters ‘*depth of water*’ (ENVO:3100031)?”.

To address this question, we used the system to query for Tara Oceans data sourced from the ‘*marine mesopelagic zone*’ (ENVO:00000213), with ‘*concentration of dioxygen in liquid water*’ (ENVO:3100011), and ‘*depth of water*’ (ENVO:3100031) values. Using those base parameters, we performed three queries for each of the three GO functional gene families. We then binned the data by oxygen concentrations into oxic and anoxic groups based on cutoff values found in the literature [109]. We then performed an indicator species analysis following the methods of Cáceres and Legendre [110] using the functional genomic GO annotations to discover

associations between the gene families and the oxic and anoxic groups. The results of the analyses, genes whose presence indicate anoxic conditions are shown in Table 1.

Notable results include ‘*anaerobic respiration*’ (GO:0009061), ‘*carbon-monoxide dehydrogenase (acceptor) activity*’ (GO:0018492), and ‘*nitrite reductase (cytochrome, ammonia-forming) activity*’ (GO:0042279). The former is expected as anaerobic respiration is required under anoxic conditions. The second GO class describes the activity of an enzyme, which plays an important role in the Wood–Ljungdahl carbon fixation pathway of anaerobic bacteria [111]. Finally, the third GO annotation describes the reduction of nitrite to ammonia, which in marine environments commonly occurs in low oxygen environments like OMZs [112,113].

These analyses, using data discovered by the system to study highly constrained ecosystems like OMZs, further demonstrate the utility of this system to enable us to get more out of our existing data. Informatic systems like the one proposed here offering granular levels of search along multiple lines of investigation (e.g., specifying environment type and physicochemical gradients along with the associated functional and taxonomic information), are needed to make sense of high-complexity ecological data.

Comparisons Across Environments

Beyond the capacity to study patterns of ecosystems sampled within an individual dataset such as those described above using the HOT 224-283 and Tara Oceans, the system is also able to facilitate broader scale comparisons between environments sampled by different projects. As our system makes use of common ontologies to integrate and harmonize data relevant to various earth and life science domains, it can enable us to ask questions that cross traditional disciplinary boundaries. To exemplify this, we used the system to investigate questions that compare the taxonomic and functional profiles of river and marine ecosystems. In our first

question we asked: “What ‘*Alphaproteobacteria*’ (NCBITaxon:28211), ‘*Archaea*’ (NCBITaxon:2157), and ‘*Verrucomicrobia*’ (NCBITaxon:74201) are most differentially abundant between surface ‘*river*’ (ENVO:00000022) and ‘*marine water body*’ (ENVO:00001999) samples, as defined by their concentration of ‘*Dissolved Inorganic Carbon*’ (PMO:00000142) (DIC)?”.

To answer these questions, we performed three system queries, one with each of the taxonomic groups, searching for samples with ‘*Dissolved Inorganic Carbon*’ (PMO:00000142) and depth values less than 30 meters. Note that we did not specify any particular dataset as we did in previous queries. This enabled the system to search through all datasets incorporated into the system for relevant data. Additionally, we binned the samples into high and low DIC groups corresponding to marine and freshwater (specifically, Amazon River) environments respectively based on cutoff values from the literature [114,115]. We used the ENVO environment types, as well as the sample’s geographic locations to verify the river and marine DIC bins. All samples from the Amazon Plume Metagenomes project labeled as being from a ‘*coastal water body*’ (ENVO:02000049) were binned into the high DIC (marine) group, except for one sample. However, that plume sample with a low DIC value, was in closest geographic proximity to the river. Hence it is likely that the sample, although just off the coast, bears a river signal. In addition to the eight-remaining high DIC Amazon Plume Metagenomes samples, the query also retrieved 13 samples from the HOT 224-283 project labeled as being from the ‘*marine wind mixed layer*’ (ENVO:01000061), as well as one sample from the BATS Chisholm project labeled as being from an ‘*ocean*’ (ENVO:00000015). All of which were binned into the high DIC marine group. All 20 samples retrieved from the Amazon River Metagenomes project in addition to the one plume sample described above were binned into the low DIC river group.

The data for each query was analyzed using an elastic net linear regression model using binomial distributions where the response variable corresponded to the marine and river DIC

bins and were plotted in Figure 7. Examining the results of these analyses, we found that from the Alphaproteobacteria phyla, two representatives of the *Pelagibacter* genus were more abundant in marine than river samples. According to the GOLD database the strains identified '*Candidatus Pelagibacter sp. FZCC0015*' (NCBITaxon:2268451) was isolated from a marine environment, while '*Candidatus Pelagibacter sp. RS39*' (NCBITaxon:1977864) was isolated from surface waters of the red sea [11]. These results confirm prior reports that *Pelagibacter* and *Candidatus Pelagibacter ubiqu*e are widely distributed and abundant in open ocean and coastal environments [116]. Additionally, the Alphaproteobacteria strain '*Nitrospirillum amazonense CBAmc*' (NCBITaxon:1441467) originally isolated from sugarcane stem in a Brazilian agrobiology field [117], was strongly associated with Amazon River samples. The strain has been studied in the context of its importance to sugarcane plant microbe interactions [118], however, its biogeography is not as well studied. Hence its abundance in the Amazon River is an example of detecting novel associations using this system.

Examining the results for Archaea, all species found in our analysis were more abundant in river than in marine environments. One Archaea species in particular, 'Candidatus Nitrosotenuis aquarius' (NCBITaxon:1846278) was significantly more abundant in river samples. The strain, an Ammonia-Oxidizing Archaeon, was originally isolated from a freshwater aquarium biofilter where it was shown to have optimal growth at 0.05% salinity [119].

Members of the Verrucomicrobia phyla are known to be abundant in freshwater [120,121] and marine [122] environments. Our results showed two Verrucomicrobia strains to be more abundant in the Amazon River than in marine environments '*Opitutus sp. GAS368*' (NCBITaxon:1882749), originally isolated from forest soil [123], and '*Nibricoccus aquaticus*' (NCBITaxon:2576891) from freshwater collected from a stream bed [124]. The remaining 4 strains including '*Coraliomargarita akajimensis DSM 45221*' (NCBITaxon:583355) isolated from

seawater sampled in the vicinity of corals [125], showed the opposite trend being more abundant in marine environments.

Turning to a final question concerning the difference in functional genomic capacities between river and marine environment we asked the question: "What '*biosynthetic process*' (GO:0009058), '*carbohydrate catabolic process*' (GO:0016052), '*carbohydrate derivative metabolic process*' (GO:1901135), and '*transmembrane transport*' (GO:0055085) genes are most different between surface '*river*' (ENVO:00000022) and '*marine water body*' (ENVO:00001999) samples as differentiated by '*Dissolved Inorganic Carbon*' (PMO:00000142) concentrations?".

To discover data by which to answer this question, we used the system with the same base query conditions described in the previous question, but instead specifying taxonomic groups we performed individual queries with each of the four GO class hierarchies. The results of the queries produced the same samples as in the previous question, but this time with subsets of their functional genomic annotations. Like with the previous question we conducted an elastic net regression analysis on the data from each GO family to determine the genes which were most differentially abundant between river and marine samples. The results are plotted in Figure 7.

Examining the '*biosynthetic process*' (GO:0009058) results, we found that genes for '*glutathione biosynthetic process*' (GO:0006750) and '*ubiquinone biosynthetic process*' (GO:0006744) were more abundant in marine than river samples. Conversely, genes for '*poly-hydroxybutyrate biosynthetic process*' (GO:0042619) and '*vitamin B6 biosynthetic process*' (GO:0042819) had the opposite trend. Both glutathione and ubiquinone are involved in both oxidative and osmotic stress responses [126–128], this could explain the result showing that the latter is increased in

saltier marine environments. Polyhydroxybutyrate (PHB) has biotechnological applications as a natural bacterially produced biopolymer [129]. As such, the result produced by the system indicating that genes for PHB production are more abundant in the Amazon River than marine environments, could be of interest when looking for where to bioprospect for new PHB producing strains.

Considering the results of the ‘*carbohydrate catabolic process*’ (GO:0016052) hierarchy, both ‘*cellulose catabolic process*’ (GO:0030245) and ‘*glucose catabolic process*’ (GO:0006007) genes were more abundant in marine environments while, ‘*xylan catabolic process*’ (GO:0045493) genes were more abundant in the river. Both glucose and cellulose are produced by algae during photosynthesis [130], which would be freely available to surface microorganisms in marine environments. Xylan on the other hand is most commonly derived from plants such as hardwoods and grasses [131]. Thus, it is logical there would be more xylan degradation in river water which contains more runoff from plants than there is in marine waters.

Some results from the analysis of the ‘*transmembrane transport*’ (GO:0055085) hierarchy showed that genes for ‘*potassium ion transmembrane transport*’ (GO:0071805) and ‘*nickel cation transmembrane transport*’ (GO:0035444) were more abundant in river samples, while genes for ‘*mercury ion transport*’ (GO:0015694) and ‘*phosphate ion transmembrane transport*’ (GO:0035435) were more abundant in marine environments. The increase in phosphate ion transport genes in marine surface samples is expected as discussed previously. Additionally, the lower abundance of genes for phosphate transport in river samples is consistent with longstanding observations that the Amazon River has elevated concentrations of phosphorus relative to the ocean [132,133].

Finally, two notable results from the '*carbohydrate derivative metabolic process*' (GO:1901135) hierarchy are that '*lipid A biosynthetic process*' (GO:0009245) was more abundant in marine samples, while '*peptidoglycan turnover*' (GO:0009254) was higher in river samples. Lipid A is known to be associated with gram-negative bacteria [134], while peptidoglycan is an essential structural component forming the outermost cell wall in gram-positive bacteria [56]. Taken together these results suggest that gram-negative bacteria are more prevalent in marine environments, whereas gram-positive bacteria are more prevalent in river environments. These results show how a hypothesis about a fundamental biogeographic question can be generated from the ontology-enriched cyberinfrastructure system presented here.

Conclusion

The presented system enables an automated method to discover and analyze data specific to new questions of biological interest. Here, we demonstrate that integrating heterogeneous data types using common vocabularies enables the search and discovery of context-dependent information. Storing searchable information computed in intensive bioinformatic workflows enables many questions to be tested with the same data corpus. Systems like this enable different investigators to ask their own unique questions on shared common datasets without needing to recompute the results themselves. Making the results of standardized data computation pipelines publicly available via semantic search capabilities not only prevents redundant data computation, but also fosters data reusability and reproducibility. The system enables queries for small subsets of the data corpus that are relevant to a specific question, and which are small enough to be computed upon with resources like a laptop computer.

The approach, however, is currently limited by the functional and taxonomic information represented within GO and NCBITaxon, as well as the limitations in the chosen computational workflow to annotate metagenomic data. Importantly, resources such as GO, Pfam, and

InterPro were created with a eukaryotic focus [16,39]. Thus, the mappings between GO and InterPro protein annotations do not as thoroughly cover prokaryotic functional genomic information. Continued efforts to map InterPro and GO annotations are needed to discover gene functions at a higher level of granularity when using the proposed workflow. Additionally, the kmer-based taxonomic identification tool Kraken2 makes use of a database of existing genomes by which to make taxonomic assignments, thus it can only identify known taxonomic groups. Finally, as the functional and taxonomic outputs are only annotated with known genes or species, it is only possible to examine the relative diversity of known genomic content.

Regarding future directions for this work, as new datasets are made available in Planet Microbe database, their functional and taxonomic annotations can be computed through the pipeline described in this manuscript. This data, along with new releases of the ontologies can be used to generate future releases of the RDF database. This would enable the systems to be used to ask and answer novel biological questions over an expanded range of ecosystems and physicochemical gradients. Additionally, the FAIR architecture for the ontology-driven harmonization and meta-analysis metagenomic datasets presented in this work could be applied in novel studies to scientific domains beyond marine and aquatic environments, enabling novel insights into microbiome of terrestrial, engineered, or host-associated environments.

All in all, this effort exemplifies a novel unified FAIR microbiome web microservice available to marine microbiologists and developers to connect to other data sources. The database, exposed via a publicly queryable API, is searchable via standardized terminology from open-source ontologies. This enables its data content to be reused by other systems and services in accordance with the vision of the FAIR principles. Future efforts to harmonize large scale microbiome datasets using commonly shared machine-readable ontologies and incorporating

them into open access cyberinfrastructure systems can enable unprecedented information sharing, discovery, and analysis.

Availability of Source Code and Requirements

Project name: Planet Microbe Semantic Web Analysis

Project home page: https://github.com/hurwitzlab/planet-microbe-semantic-web-analysis

Operating system(s): Platform independent

Programming language: Python 3.8.5+

Other requirements: R 4.2.2, Apache Jena TBD2 4.3.2, Apache Jena Fuseki2 4.3.2, ROBOT 1.8.3+, Java 11+

License: The MIT License (MIT)

RRID:SCR_024478

biotools:planet-microbe

Project name: Planet Microbe Functional Annotation

Project home page: https://github.com/hurwitzlab/planet-microbe-functional-annotation/

Operating system(s): Linux

Programming language: SLURM/Linux Shell

Other requirements: Python3 3.7+ miniconda3 3.7-23.1.0, Java jdk-11.0.8+, bowtie2 2.4.2, Trimmomatic 0.39, vsearch 2.21.1, Kraken2, FragGeneScan 1.31, InterProScan 5.46-81.0

License: The MIT License (MIT)

Data Availability

The code for the functional and taxonomic metagenomic annotation pipeline used in this work is available from the following GitHub repository [28]. The data sets supporting the results of this article are available from the Zenodo data repository [40]. The scripts used for 1) creating the Semantic Web data integration pipeline, 2) querying the public API, and 3) analyzing the results of the biological questions discussed in the manuscript are available from the following GitHub repository [44]. Tutorials for setting up custom queries as well as analyzing the results derived from the queries can be found at the following protocols.io page [26]. An archival copy of the code and supporting data including all data queried from the RDF server and analyzed in the manuscript is available via the GigaScience database GigaDB [135].

Abbreviations

EBI: European Bioinformatics Institute; ENVO: Environment Ontology; GO: Gene Ontology; HOT: Hawaiian Ocean Time; HPC: High Performance Computing; MIxS: Minimum Information about any (x) Sequence; NCBI: National Center for Biotechnology Information; NCBITaxon: NCBI Organismal Taxonomy Database as an Ontology; OBO: Open Biomedical and Biological Ontologies; OMZs: Oxygen Minimum Zones; ORFs: Open Reading Frames; OWL: Ontology Web Language; PMO: Planet Microbe Ontology; RDF: Resource Description Framework; WGS: Whole Genome Sequencing; FAIR: Findable Accessible Interoperable, and Reusable.

ACKNOWLEDGEMENTS

We would like to thank Dr. Pier L. Buttigieg and Professor Antje Boetius for helping foster the original idea for the ontology-based data discovery workflow that we built upon in this paper. We

would like to thank Dr. Peter Winstanley for the Semantic Web tooling suggestions used in this work. We also thank Dr. James Overton for allowing us to reuse a portion of an RDF graph structure he created. We would also like to thank Adam Michel from the University of Arizona IT services for helping us make our new database's API publicly accessible. We also thank Matthew Bomhoff for providing example code for querying the Planet Microbe Database's API. We would also like to thank Heidi Steiner and the University of Arizona Sarver Heart Center for funding the publication of this work. Finally, we would like to thank Chris Mungall, as well as members of the Hurwitz Lab for their feedback and discussions of the work.

Competing Interests

BH holds concurrent appointments as an Associate Professor of Biosystems Engineering at the University Arizona and as an Amazon Scholar. This publication describes work performed at the University Arizona and is not associated with Amazon. The remaining authors declare that the research was conducted in the absence of any commercial or financial relationships that could be construed as a potential conflict of interest.

FUNDING

This work was supported by the National Science Foundation [OCE-1639614 Planet Microbe to B.L.H., in part]; National Science Foundation [CISE-1640775 Ocean Cloud Commons to B.L.H., in part]; the Simons Foundation muSCOPE [ID 481471 to B.L.H.]; the Gordon and Betty Moore Foundation [GBMF 8751 to B.L.H.]; and the Academy of Finland [ID 339172 to AP]; Funding for open access charge: Sarver Heart Center's Finley and Florence Brown Endowed Research Award.

Authors' Contributions

K.B.: Running functional analysis pipeline, creation of RDF database and codebase, conducting analyses and writing the original draft. M.M: writing the functional analysis pipeline code. A.P: Method validation, supervision, reviewing and editing. B.H: Project coordination, supervision, administration, grant writing, reviewing, and editing.

References

1. Turnbaugh PJ, Ley RE, Hamady M, Fraser-Liggett CM, Knight R, Gordon JI. The Human Microbiome Project. *Nature*. 2007; doi: 10.1038/nature06244.
2. Gilbert JA, Meyer F, Antonopoulos D, Balaji P, Brown CT, Brown CT, et al.. Meeting Report: The Terabase Metagenomics Workshop and the Vision of an Earth Microbiome Project. *Stand Genomic Sci*. 2010; doi: 10.4056/sigs.1433550.
3. Sunagawa S, Coelho LP, Chaffron S, Kultima JR, Labadie K, Salazar G, et al.. Structure and function of the global ocean microbiome. *Science*. 2015; doi: 10.1126/science.1261359.
4. DeLong EF, Karl DM. Genomic perspectives in microbial oceanography. *Nature*. Nature Publishing Group; 2005; doi: 10.1038/nature04157.
5. Graham EB, Knelman JE, Schindlbacher A, Siciliano S, Breulmann M, Yannarell A, et al.. Microbes as Engines of Ecosystem Function: When Does Community Structure Enhance Predictions of Ecosystem Processes? *Front Microbiol*. Frontiers; 2016; doi: 10.3389/fmicb.2016.00214.
6. Gao C, Fernandez VI, Lee KS, Fenizia S, Pohnert G, Seymour JR, et al.. Single-cell bacterial transcription measurements reveal the importance of dimethylsulfoniopropionate (DMSP) hotspots in ocean sulfur cycling. *Nat Commun*. Nature Publishing Group; 2020; doi: 10.1038/s41467-020-15693-z.
7. Wilkinson MD, Dumontier M, Aalbersberg IjJ, Appleton G, Axton M, Baak A, et al.. The FAIR Guiding Principles for scientific data management and stewardship. *Sci Data*. 2016; doi: 10.1038/sdata.2016.18.
8. Meyer F, Paarmann D, D’Souza M, Olson R, Glass E, Kubal M, et al.. The metagenomics RAST server – a public resource for the automatic phylogenetic and functional analysis of metagenomes. *BMC Bioinformatics*. 2008; doi: 10.1186/1471-2105-9-386.
9. Caporaso JG, Kuczynski J, Stombaugh J, Bittinger K, Bushman FD, Costello EK, et al.. QIIME allows analysis of high-throughput community sequencing data. *Nat Methods*. 2010; doi: 10.1038/nmeth.f.303.
10. Mitchell AL, Almeida A, Beracochea M, Boland M, Burgin J, Cochrane G, et al.. MGnify: the microbiome analysis resource in 2020. *Nucleic Acids Res*. 2020; doi: 10.1093/nar/gkz1035.
11. Mukherjee S, Stamatis D, Bertsch J, Ovchinnikova G, Sundaramurthi JC, Lee J, et al.. Genomes OnLine Database (GOLD) v.8: overview and updates. *Nucleic Acids Res*. 2021; doi: 10.1093/nar/gkaa983.
12. Yilmaz P, Kottmann R, Field D, Knight R, Cole JR, Amaral-Zettler L, et al.. Minimum information about a marker gene sequence (MIMARKS) and minimum information about any (x) sequence (MIxS) specifications. *Nat Biotechnol*. 2011; doi: 10.1038/nbt.1823.
13. Wood-Charlson EM, Anubhav, Auberry D, Blanco H, Borkum MI, Corilo YE, et al.. The National Microbiome Data Collaborative: enabling microbiome science. *Nat Rev Microbiol*.

Nature Publishing Group; 2020; doi: 10.1038/s41579-020-0377-0.
14. Smith B, Ashburner M, Rosse C, Bard J, Bug W, Ceusters W, et al.. The OBO Foundry: coordinated evolution of ontologies to support biomedical data integration. *Nat Biotechnol*. Nature Publishing Group; 2007; doi: 10.1038/nbt1346.
15. Walls RL, Deck J, Guralnick R, Baskauf S, Beaman R, Blum S, et al.. Semantics in Support of Biodiversity Knowledge Discovery: An Introduction to the Biological Collections Ontology and Related Ontologies. Bajic VB, editor. *PLoS ONE*. 2014; doi: 10.1371/journal.pone.0089606.
16. Ashburner M, Ball CA, Blake JA, Botstein D, Butler H, Cherry JM, et al.. Gene Ontology: tool for the unification of biology. *Nat Genet*. 2000; doi: 10.1038/75556.
17. Blake JA, Bult CJ. Beyond the data deluge: Data integration and bio-ontologies. *J Biomed Inform*. 2006; doi: 10.1016/j.jbi.2006.01.003.
18. Buttigieg P, Morrison N, Smith B, Mungall CJ, Lewis SE, the ENVO Consortium. The environment ontology: contextualising biological and biomedical entities. *J Biomed Semant*. 2013; doi: 10.1186/2041-1480-4-43.
19. Buttigieg PL, Pafilis E, Lewis SE, Schildhauer MP, Walls RL, Mungall CJ. The environment ontology in 2016: bridging domains with increased scope, semantic density, and interoperation. *J Biomed Semant*. 2016; doi: 10.1186/s13326-016-0097-6.
20. Federhen S. The NCBI Taxonomy database. *Nucleic Acids Res*. 2012; doi: 10.1093/nar/gkr1178.
21. Ponsero AJ, Bomhoff M, Blumberg K, Youens-Clark K, Herz NM, Wood-Charlson EM, et al.. Planet Microbe: a platform for marine microbiology to discover and analyze interconnected ‘omics and environmental data. *Nucleic Acids Res*. 2021; doi: 10.1093/nar/gkaa637.
22. Blumberg KL, Ponsero AJ, Bomhoff M, Wood-Charlson EM, DeLong EF, Hurwitz BL. Ontology-Enriched Specifications Enabling Findable, Accessible, Interoperable, and Reusable Marine Metagenomic Datasets in Cyberinfrastructure Systems. *Front Microbiol*. 122021;
23. Galperin MY, Makarova KS, Wolf YI, Koonin EV. Expanded microbial genome coverage and improved protein family annotation in the COG database. *Nucleic Acids Res*. 2015; doi: 10.1093/nar/gku1223.
24. Kanehisa M, Goto S. KEGG: Kyoto Encyclopedia of Genes and Genomes. *Nucleic Acids Res*. 28:27–302000;
25. Overbeek R, Begley T, Butler RM, Choudhuri JV, Chuang H-Y, Cohoon M, et al.. The subsystems approach to genome annotation and its use in the project to annotate 1000 genomes. *Nucleic Acids Res*. 2005; doi: 10.1093/nar/gki866.
26. Blumberg K, Ponsero A, Hurwitz B. Planet Microbe Semantic Web Application V.2. protocols.io. 2023 http://dx.doi.org/10.17504/protocols.io.e6nvwkw19vmk/v2.
27. Ponsero A: Planet Microbe. 2020. https://www.planetmicrobe.org. Accessed 22 Jan 2022.
28. Miller M: Planet Microbe Functional Annotation. *Github*. https://github.com/hurwitzlab/planet-microbe-functional-annotation. Accessed 6 July 2023.
29. Langmead B, Trapnell C, Pop M, Salzberg SL. Ultrafast and memory-efficient alignment of short DNA sequences to the human genome. *Genome Biol*. 2009; doi: 10.1186/gb-2009-10-3-r25.
30. Bolger AM, Lohse M, Usadel B. Trimmomatic: a flexible trimmer for Illumina sequence data. *Bioinformatics*. 2014; doi: 10.1093/bioinformatics/btu170.
31. Rognes T, Flouri T, Nichols B, Quince C, Mahé F. VSEARCH: a versatile open source tool for metagenomics. *PeerJ*. 2016; doi: 10.7717/peerj.2584.
32. Wood DE, Lu J, Langmead B. Improved metagenomic analysis with Kraken 2. *Genome Biol*. 2019; doi: 10.1186/s13059-019-1891-0.
33. Langmead B: Kraken 2, KrakenUniq and Bracken indexes. *Github*. https://benlangmead.github.io/aws-indexes/k2. Accessed 4 Aug 2022.
34. EMBL’s European Bioinformatics Institute (EMBL-EBI): MGnify Pipeline 4.1. 2021. https://www.ebi.ac.uk/metagenomics/pipelines/4.1. Accessed 19 May 2021.

35. Rho M, Tang H, Ye Y. FragGeneScan: predicting genes in short and error-prone reads. *Nucleic Acids Res*. 2010; doi: 10.1093/nar/gkq747.
36. Hyatt D, Chen G-L, Locascio PF, Land ML, Larimer FW, Hauser LJ. Prodigal: prokaryotic gene recognition and translation initiation site identification. *BMC Bioinformatics*. 2010; doi: 10.1186/1471-2105-11-119.
37. Zdobnov EM, Apweiler R. InterProScan--an integration platform for the signature-recognition methods in InterPro. *Bioinforma Oxf Engl*. 2001; doi: 10.1093/bioinformatics/17.9.847.
38. Quevillon E, Silventoinen V, Pillai S, Harte N, Mulder N, Apweiler R, et al.. InterProScan: protein domains identifier. *Nucleic Acids Res*. 2005; doi: 10.1093/nar/gki442.
39. Bateman A, Birney E, Durbin R, Eddy SR, Howe KL, Sonnhammer EL. The Pfam protein families database. *Nucleic Acids Res*. 2000; doi: 10.1093/nar/28.1.263.
40. Blumberg K, Ponsero A, Miller M, Hurwitz B. Planet Microbe Functional and Taxonomic annotation of Illumina WGS Prokaryotic Fraction for Semantic Web Analysis. Zenodo. 2023. https://doi.org/10.5281/zenodo.7732330.
41. Apache Jena TDB2. 2022. https://jena.apache.org/documentation/tdb2. Accessed 28 Jan 2022.
42. Blumberg K: Planet Microbe Ontology. *Github*. https://github.com/hurwitzlab/planet-microbe-ontology. Accessed 26 Jan 2022.
43. Jackson RC, Balhoff JP, Douglass E, Harris NL, Mungall CJ, Overton JA. ROBOT: A Tool for Automating Ontology Workflows. *BMC Bioinformatics*. 2019; doi: 10.1186/s12859-019-3002-3.
44. Blumberg K: Planet Microbe Semantic Web Analysis. *Github*. https://github.com/hurwitzlab/planet-microbe-semantic-web-analysis. Accessed 6 Jun 2023.
45. Ponsero A: Planet Microbe Search API. 2020. https://www.planetmicrobe.org/api/search. Accessed 10 Feb 2022.
46. Bomhoff M: Planet Microbe App. 2020. https://github.com/hurwitzlab/planet-microbe-app. Accessed 10 Feb 2022.
47. Gloor GB, Macklaim JM, Pawlowsky-Glahn V, Egozcue JJ. Microbiome Datasets Are Compositional: And This Is Not Optional. *Front Microbiol*. 2017; doi: 10.3389/fmicb.2017.02224.
48. Friedman J, Hastie T, Tibshirani R. Regularization Paths for Generalized Linear Models via Coordinate Descent. *J Stat Softw*. 2010; doi: 10.18637/jss.v033.i01.
49. Mende DR, Bryant JA, Aylward FO, Eppley JM, Nielsen T, Karl DM, et al.. Environmental drivers of a microbial genomic transition zone in the ocean’s interior. *Nat Microbiol*. Nature Publishing Group; 2017; doi: 10.1038/s41564-017-0008-3.
50. Karl DM, Lukas R. The Hawaii Ocean Time-series (HOT) program: Background, rationale and field implementation. *Deep Sea Res Part II Top Stud Oceanogr*. 1996; doi: 10.1016/0967-0645(96)00005-7.
51. Bryant JA, Aylward FO, Eppley JM, Karl DM, Church MJ, DeLong EF. Wind and sunlight shape microbial diversity in surface waters of the North Pacific Subtropical Gyre. *ISME J*. 2016; doi: 10.1038/ismej.2015.221.
52. Johnson ZI, Zinser ER, Coe A, McNulty NP, Woodward EMS, Chisholm SW. Niche Partitioning Among Prochlorococcus Ecotypes Along Ocean-Scale Environmental Gradients. *Science*. American Association for the Advancement of Science; 2006; doi: 10.1126/science.1118052.
53. Flombaum P, Gallegos JL, Gordillo RA, Rincón J, Zabala LL, Jiao N, et al.. Present and future global distributions of the marine Cyanobacteria Prochlorococcus and Synechococcus. *Proc Natl Acad Sci U S A*. 2013; doi: 10.1073/pnas.1307701110.
54. Muñoz-Marín MC, Gómez-Baena G, López-Lozano A, Moreno-Cabezuelo JA, Díez J, García-Fernández JM. Mixotrophy in marine picocyanobacteria: use of organic compounds by Prochlorococcus and Synechococcus. *ISME J*. Nature Publishing Group; 2020; doi: 10.1038/s41396-020-0603-9.

55. Biller SJ, Berube PM, Berta-Thompson JW, Kelly L, Roggensack SE, Awad L, et al.. Genomes of diverse isolates of the marine cyanobacterium Prochlorococcus. *Sci Data*. 2014; doi: 10.1038/sdata.2014.34.
56. Madigan MT, editor. Brock biology of microorganisms. 13th ed. San Francisco: Benjamin Cummings;
57. Morel A. Light and marine photosynthesis: a spectral model with geochemical and climatological implications. *Prog Oceanogr*. 1991; doi: https://doi.org/10.1016/0079-6611(91)90004-6.
58. Guerrero-Cruz S, Vaksmaa A, Horn MA, Niemann H, Pijuan M, Ho A. Methanotrophs: Discoveries, Environmental Relevance, and a Perspective on Current and Future Applications. *Front Microbiol*. 2021; doi: 10.3389/fmicb.2021.678057.
59. Reeburgh WS, Heggie DT. Microbial methane consumption reactions and their effect on methane distributions in freshwater and marine environments1. *Limnol Oceanogr*. 1977; doi: 10.4319/lo.1977.22.1.0001.
60. Hoehler T, Losey NA, Gunsalus RP, McInerney MJ. Environmental Constraints that Limit Methanogenesis. In: Stams AJM, Sousa D, editors. *Biog Hydrocarb*. Cham: Springer International Publishing;
61. Kuo P-A, Kuo C-H, Lai Y-K, Graumann PL, Tu J. Phosphate limitation induces the intergeneric inhibition of Pseudomonas aeruginosa by Serratia marcescens isolated from paper machines. *FEMS Microbiol Ecol*. 2013; doi: 10.1111/1574-6941.12086.
62. Lin S, Litaker RW, Sunda WG. Phosphorus physiological ecology and molecular mechanisms in marine phytoplankton. *J Phycol*. 2016; doi: 10.1111/jpy.12365.
63. Jb C, Jw A, Er P, E B. Phosphorus-limited bacterioplankton growth in the Sargasso Sea. *Aquat Microb Ecol*. 1997; doi: 10.3354/ame013141.
64. Wu J, Sunda W, Boyle EA, Karl DM. Phosphate Depletion in the Western North Atlantic Ocean. *Science*. American Association for the Advancement of Science; 2000; doi: 10.1126/science.289.5480.759.
65. Thingstad TF, Krom MD, Mantoura RFC, Flaten GAF, Groom S, Herut B, et al.. Nature of Phosphorus Limitation in the Ultraoligotrophic Eastern Mediterranean. *Science*. American Association for the Advancement of Science; 2005; doi: 10.1126/science.1112632.
66. Duhamel S, Diaz JM, Adams JC, Djaoudi K, Steck V, Waggoner EM. Phosphorus as an integral component of global marine biogeochemistry. *Nat Geosci*. Nature Publishing Group; 2021; doi: 10.1038/s41561-021-00755-8.
67. Sunda W. Feedback Interactions between Trace Metal Nutrients and Phytoplankton in the Ocean. *Front Microbiol*. 32012;
68. Letelier RM, Björkman KM, Church MJ, Hamilton DS, Mahowald NM, Scanza RA, et al.. Climate-driven oscillation of phosphorus and iron limitation in the North Pacific Subtropical Gyre. *Proc Natl Acad Sci*. Proceedings of the National Academy of Sciences; 2019; doi: 10.1073/pnas.1900789116.
69. Dittrich M, Sibler S. Calcium carbonate precipitation by cyanobacterial polysaccharides. 2010; doi: 10.1144/SP336.4.
70. Xu H, Peng X, Bai S, Ta K, Yang S, Liu S, et al.. Precipitation of calcium carbonate mineral induced by viral lysis of cyanobacteria: evidence from laboratory experiments. *Biogeosciences*. Copernicus GmbH; 2019; doi: 10.5194/bg-16-949-2019.
71. Morel FMM, Price NM. The Biogeochemical Cycles of Trace Metals in the Oceans. *Science*. American Association for the Advancement of Science; 2003; doi: 10.1126/science.1083545.
72. Sclater FR, Boyle E, Edmond JM. On the marine geochemistry of nickel. *Earth Planet Sci Lett*. 1976; doi: 10.1016/0012-821X(76)90103-5.
73. Archer C, Vance D, Milne A, Lohan MC. The oceanic biogeochemistry of nickel and its isotopes: New data from the South Atlantic and the Southern Ocean biogeochemical divide. *Earth Planet Sci Lett*. 2020; doi: 10.1016/j.epsl.2020.116118.

74. Morel FMM, Kustka AB, Shaked Y. The role of unchelated Fe in the iron nutrition of phytoplankton. *Limnol Oceanogr*. 2008; doi: 10.4319/lo.2008.53.1.0400.
75. Tagliabue A, Bowie AR, Boyd PW, Buck KN, Johnson KS, Saito MA. The integral role of iron in ocean biogeochemistry. *Nature*. Nature Publishing Group; 2017; doi: 10.1038/nature21058.
76. Liu X, Millero FJ. The solubility of iron in seawater. *Mar Chem*. 2002; doi: 10.1016/S0304-4203(01)00074-3.
77. Croot PL, Bowie AR, Frew RD, Maldonado MT, Hall JA, Safi KA, et al.. Retention of dissolved iron and FeII in an iron induced Southern Ocean phytoplankton bloom. *Geophys Res Lett*. 2001; doi: 10.1029/2001GL013023.
78. Moffett JW, Goepfert TJ, Naqvi SWA. Reduced iron associated with secondary nitrite maxima in the Arabian Sea. *Deep Sea Res Part Oceanogr Res Pap*. 2007; doi: 10.1016/j.dsr.2007.04.004.
79. Boyle EA, Bergquist BA, Kayser RA, Mahowald N. Iron, manganese, and lead at Hawaii Ocean Time-series station ALOHA: Temporal variability and an intermediate water hydrothermal plume. *Geochim Cosmochim Acta*. 2005; doi: 10.1016/j.gca.2004.07.034.
80. Flynn KJ, Wright CRN. The simultaneous assimilation of ammonium and l-arginine by the marine diatom Phaeodactylum tricornutum Bohlin. *J Exp Mar Biol Ecol*. 1986; doi: 10.1016/0022-0981(86)90258-3.
81. Ouverney CC, Fuhrman JA. Marine planktonic archaea take up amino acids. *Appl Environ Microbiol*. 2000; doi: 10.1128/AEM.66.11.4829-4833.2000.
82. Teira E, van Aken H, Veth C, Herndl GJ. Archaeal uptake of enantiomeric amino acids in the meso- and bathypelagic waters of the North Atlantic. *Limnol Oceanogr*. 2006; doi: 10.4319/lo.2006.51.1.0060.
83. Zubkov MV, Tarran GA, Fuchs BM. Depth related amino acid uptake by Prochlorococcus cyanobacteria in the Southern Atlantic tropical gyre. *FEMS Microbiol Ecol*. 2004; doi: 10.1016/j.femsec.2004.06.009.
84. Rocap G, Larimer FW, Lamerdin J, Malfatti S, Chain P, Ahlgren NA, et al.. Genome divergence in two Prochlorococcus ecotypes reflects oceanic niche differentiation. *Nature*. 2003; doi: 10.1038/nature01947.
85. Kettler GC, Martiny AC, Huang K, Zucker J, Coleman ML, Rodrigue S, et al.. Patterns and Implications of Gene Gain and Loss in the Evolution of Prochlorococcus. *PLoS Genet*. 2007; doi: 10.1371/journal.pgen.0030231.
86. Zinser ER, Johnson ZI, Coe A, Karaca E, Veneziano D, Chisholm SW. Influence of light and temperature on Prochlorococcus ecotype distributions in the Atlantic Ocean. *Limnol Oceanogr*. 2007; doi: 10.4319/lo.2007.52.5.2205.
87. Garczarek L, Partensky F. Expression and phylogeny of the multiple antenna genes of the low-light-adapted strain Prochlorococcus marinus SS120 (Oxyphotobacteria). :11.
88. Aldunate M, De la Iglesia R, Bertagnolli AD, Ulloa O. Oxygen modulates bacterial community composition in the coastal upwelling waters off central Chile. *Deep Sea Res Part II Top Stud Oceanogr*. 2018; doi: 10.1016/j.dsr2.2018.02.001.
89. Sun Q, Song J, Li X, Yuan H, Wang Q. The bacterial diversity and community composition altered in the oxygen minimum zone of the Tropical Western Pacific Ocean. *J Oceanol Limnol*. 2021; doi: 10.1007/s00343-021-0370-0.
90. Long AM, Jurgensen SK, Petchel AR, Savoie ER, Brum JR. Microbial Ecology of Oxygen Minimum Zones Amidst Ocean Deoxygenation. *Front Microbiol*. 2021; doi: 10.3389/fmicb.2021.748961.
91. Paulmier A, Ruiz-Pino D. Oxygen minimum zones (OMZs) in the modern ocean. *Prog Oceanogr*. 2009; doi: 10.1016/j.pocean.2008.08.001.
92. Stramma L, Johnson GC, Sprintall J, Mohrholz V. Expanding Oxygen-Minimum Zones in the Tropical Oceans. *Science*. American Association for the Advancement of Science; 2008; doi: 10.1126/science.1153847.

93. Stramma L, Prince ED, Schmidtko S, Luo J, Hoolihan JP, Visbeck M, et al.. Expansion of oxygen minimum zones may reduce available habitat for tropical pelagic fishes. *Nat Clim Change*. Nature Publishing Group; 2012; doi: 10.1038/nclimate1304.
94. Lalli CM, Parsons TR. Biological oceanography: an introduction. 2nd ed. Oxford [England]: Butterworth Heinemann;
95. Stevens H, Ulloa O. Bacterial diversity in the oxygen minimum zone of the eastern tropical South Pacific. *Environ Microbiol*. 2008; doi: 10.1111/j.1462-2920.2007.01539.x.
96. Hawley AK, Brewer HM, Norbeck AD, Paša-Tolić L, Hallam SJ. Metaproteomics reveals differential modes of metabolic coupling among ubiquitous oxygen minimum zone microbes. *Proc Natl Acad Sci*. Proceedings of the National Academy of Sciences; 2014; doi: 10.1073/pnas.1322132111.
97. Rajpathak SN, Banerjee R, Mishra PG, Khedkar AM, Patil YM, Joshi SR, et al.. An exploration of microbial and associated functional diversity in the OMZ and non-OMZ areas in the Bay of Bengal. *J Biosci*. 43:635–482018;
98. Fernandes GL, Shenoy BD, Damare SR. Diversity of Bacterial Community in the Oxygen Minimum Zones of Arabian Sea and Bay of Bengal as Deduced by Illumina Sequencing. *Front Microbiol*. 102020;
99. Du H, Zhang W, Zhang W, Zhang W, Pan H, Pan Y, et al.. Magnetosome Gene Duplication as an Important Driver in the Evolution of Magnetotaxis in the Alphaproteobacteria. *mSystems*. American Society for Microbiology; doi: 10.1128/mSystems.00315-19.
100. Du H, Zhang W, Lin W, Pan H, Xiao T, Wu L-F. Genomic analysis of a pure culture of magnetotactic bacterium Terasakiella sp. SH-1. *J Oceanol Limnol*. 2021; doi: 10.1007/s00343-021-1054-5.
101. Marx CJ, Bringel F, Chistoserdova L, Moulin L, Farhan Ul Haque M, Fleischman DE, et al.. Complete Genome Sequences of Six Strains of the Genus Methylobacterium. *J Bacteriol*. 2012; doi: 10.1128/JB.01009-12.
102. Ueki A, Akasaka H, Suzuki D, Ueki K 2006. Paludibacter propionicigenes gen. nov., sp. nov., a novel strictly anaerobic, Gram-negative, propionate-producing bacterium isolated from plant residue in irrigated rice-field soil in Japan. *Int J Syst Evol Microbiol*. Microbiology Society,; doi: 10.1099/ijs.0.63896-0.
103. Reimer LC, Sarda Carbasse J, Koblitz J, Podstawka A, Overmann J. Maridesulfovibrio salexigens (Postgate and Campbell 1966) Waite et al. 2020. DSMZ;
104. Henkel JV, Dellwig O, Pollehne F, Herlemann DPR, Leipe T, Schulz-Vogt HN. A bacterial isolate from the Black Sea oxidizes sulfide with manganese(IV) oxide. *Proc Natl Acad Sci*. Proceedings of the National Academy of Sciences; 2019; doi: 10.1073/pnas.1906000116.
105. Sikorski J, Lapidus A, Copeland A, Glavina Del Rio T, Nolan M, Lucas S, et al.. Complete genome sequence of Sulfurospirillum deleyianum type strain (5175T). *Stand Genomic Sci*. 2010; doi: 10.4056/sigs.671209.
106. Dicks LMT, Holzapfel WilhelmH, Satomi M, Kimura B, Fujii T. Tetragenococcus. *Bergeys Man Syst Archaea Bact*. John Wiley & Sons, Ltd;
107. Graf J, editor. Aeromonas. Norfolk, UK: Caister Academic Press;
108. Pradel N, Fardeau M-L, Tindall BJ, Spring S. Anaerohalosphaera lusitana gen. nov., sp. nov., and Limihaloglobus sulfuriphilus gen. nov., sp. nov., isolated from solar saltern sediments, and proposal of Anaerohalosphaeraceae fam. nov. within the order Sedimentisphaerales. *Int J Syst Evol Microbiol*. 2020; doi: 10.1099/ijsem.0.003919.
109. Orsi W, Song YC, Hallam S, Edgcomb V. Effect of oxygen minimum zone formation on communities of marine protists. *ISME J*. Nature Publishing Group; 2012; doi: 10.1038/ismej.2012.7.
110. Cáceres MD, Legendre P. Associations between species and groups of sites: indices and statistical inference. *Ecology*. 2009; doi: 10.1890/08-1823.1.
111. Mander LN, Liu H. Comprehensive natural products II: chemistry and biology. Oxford:

Elsevier Science;
112. Kamp A, de Beer D, Nitsch JL, Lavik G, Stief P. Diatoms respire nitrate to survive dark and anoxic conditions. *Proc Natl Acad Sci*. Proceedings of the National Academy of Sciences; 2011; doi: 10.1073/pnas.1015744108.
113. Kamp A, Stief P, Knappe J, Beer D de. Response of the Ubiquitous Pelagic Diatom Thalassiosira weissflogii to Darkness and Anoxia. *PLOS ONE*. Public Library of Science; 2013; doi: 10.1371/journal.pone.0082605.
114. Land PE, Findlay HS, Shutler JD, Ashton IGC, Holding T, Grouazel A, et al.. Optimum satellite remote sensing of the marine carbonate system using empirical algorithms in the global ocean, the Greater Caribbean, the Amazon Plume and the Bay of Bengal. *Remote Sens Environ*. 2019; doi: 10.1016/j.rse.2019.111469.
115. Yan J, Lin Q, Poh SC, Li Y, Zhan L. Underway Measurement of Dissolved Inorganic Carbon (DIC) in Estuarine Waters. *J Mar Sci Eng*. Multidisciplinary Digital Publishing Institute; 2020; doi: 10.3390/jmse8100765.
116. Zhao Y, Temperton B, Thrash JC, Schwalbach MS, Vergin KL, Landry ZC, et al.. Abundant SAR11 viruses in the ocean. *Nature*. Nature Publishing Group; 2013; doi: 10.1038/nature11921.
117. Schwab S, Terra LA, Baldani JI. Genomic characterization of Nitrospirillum amazonense strain CBAmC, a nitrogen-fixing bacterium isolated from surface-sterilized sugarcane stems. *Mol Genet Genomics*. 2018; doi: 10.1007/s00438-018-1439-0.
118. Terra LA, de Soares CP, Meneses CHSG, Tadra Sfeir MZ, de Souza EM, Silveira V, et al.. Transcriptome and proteome profiles of the diazotroph Nitrospirillum amazonense strain CBAmC in response to the sugarcane apoplast fluid. *Plant Soil*. 2020; doi: 10.1007/s11104-019-04201-y.
119. Sauder LA, Engel K, Lo C-C, Chain P, Neufeld JD. “Candidatus Nitrosotenuis aquarius,” an Ammonia-Oxidizing Archaeon from a Freshwater Aquarium Biofilter. *Appl Environ Microbiol*. 2018; doi: 10.1128/AEM.01430-18.
120. Zwart G, van Hannen EJ, Kamst-van Agterveld MP, Van der Gucht K, Lindström ES, Van Wichelen J, et al.. Rapid screening for freshwater bacterial groups by using reverse line blot hybridization. *Appl Environ Microbiol*. 2003; doi: 10.1128/AEM.69.10.5875-5883.2003.
121. Newton RJ, Jones SE, Eiler A, McMahon KD, Bertilsson S. A guide to the natural history of freshwater lake bacteria. *Microbiol Mol Biol Rev MMBR*. 2011; doi: 10.1128/MMBR.00028-10.
122. Freitas S, Hatosy S, Fuhrman JA, Huse SM, Welch DBM, Sogin ML, et al.. Global distribution and diversity of marine Verrucomicrobia. *ISME J*. 2012; doi: 10.1038/ismej.2012.3.
123. He S, Stevens SLR, Chan L-K, Bertilsson S, Glavina del Rio T, Tringe SG, et al.. Ecophysiology of Freshwater Verrucomicrobia Inferred from Metagenome-Assembled Genomes. *mSphere*. 2017; doi: 10.1128/mSphere.00277-17.
124. Baek K, Song J, Cho J-C, Chung EJ, Choi A. Nibricoccus aquaticus gen. nov., sp. nov., a new genus of the family Opitutaceae isolated from hyporheic freshwater. *Int J Syst Evol Microbiol*. 2019; doi: 10.1099/ijsem.0.003198.
125. Mavromatis K, Abt B, Brambilla E, Lapidus A, Copeland A, Deshpande S, et al.. Complete genome sequence of Coraliomargarita akajimensis type strain (04OKA010-24T). *Stand Genomic Sci*. BioMed Central; 2010; doi: 10.4056/sigs.952166.
126. Smirnova GV, Oktyabrsky ON. Glutathione in bacteria. *Biochem Biokhimiia*. 2005; doi: 10.1007/s10541-005-0248-3.
127. Masip L, Veeravalli K, Georgiou G. The Many Faces of Glutathione in Bacteria. *Antioxid Redox Signal*. Mary Ann Liebert, Inc., publishers; 2006; doi: 10.1089/ars.2006.8.753.
128. Saini R. Coenzyme Q10: The essential nutrient. *J Pharm Bioallied Sci*. 2011; doi: 10.4103/0975-7406.84471.
129. Utsunomia C, Ren Q, Zinn M. Poly(4-Hydroxybutyrate): Current State and Perspectives. *Front Bioeng Biotechnol*. 2020; doi: 10.3389/fbioe.2020.00257.
130. Samiee S, Ahmadzadeh H, Hosseini M, Lyon S. Chapter 17 - Algae as a Source of

Microcrystalline Cellulose. In: Hosseini M, editor. *Adv Bioprocess Altern Fuels Biobased Chem Bioprod*. Woodhead Publishing;
131. Himmel ME. Direct microbial conversion of biomass to advanced biofuels. Amsterdam: Elsevier;
132. Chase EM, Sayles FL. Phosphorus in suspended sediments of the Amazon River. *Estuar Coast Mar Sci*. 1980; doi: 10.1016/S0302-3524(80)80063-6.
133. Rao J-L, Berner RA. Phosphorus dynamics in the Amazon river and estuary. *Chem Geol*. 1993; doi: 10.1016/0009-2541(93)90218-8.
134. Raetz CRH, Whitfield C. Lipopolysaccharide Endotoxins. *Annu Rev Biochem*. 2002; doi: 10.1146/annurev.biochem.71.110601.135414.
135. Kai B, Matthew M, Alise P, Bonnie HL. Supporting data for "Ontology-Driven Analysis of Marine Metagenomics: What More Can We Learn from Our Data?" GigaScience Database. 2023. http://dx.doi.org/10.5524/102419.
136. OBO Technical WG: Open Biological and Biomedical Ontology Foundry. 2022. https://obofoundry.org. Accessed 31 May 2023.

Table and Figure Legends

Figure 1: (A) Depth profiles of '*Synechococcus*' (NCBITaxon:1129) and '*Prochlorococcus*' (NCBITaxon:1218) reads from the HOT 224-283 dataset. Plots show the summation of relativized counts of all taxonomic assignments made at the genus taxonomic level and below for both genera respectively on the X-axis. The Y-axis shows the water column depth. (B) Depth profiles of '*photosynthesis*' (GO:0015979) and '*methanogenesis*' (GO:0015948) gene relative abundance in metagenomic samples from the HOT 224-283 dataset. (C) Depth profiles of '*phosphate ion binding*' (GO:0042301) gene relative abundance and accompanying measurements of water column '*concentration of phosphate in liquid water*' (ENVO:3100026) respectively, from the HOT 224-283 dataset.

Figure 2: HOT 224-283 Cation Binding Gene Elastic Net Linear Regression Against Depth Genes resulting from an elastic net linear regression analysis for feature selection performed on the '*cation binding*' (GO:0043169) gene family and sample depths in HOT Aloha 224-283 samples. Results are colored by depth bins for shallow, intermediate, and deep depth ranges. The X-axis shows gene count values that are normalized by the number of ORFs, as well as Aitchison (centered log ratio) transformed.

Figure 3: HOT 224-283 Depth profiles of Low Light Adapted *Cyanobacteria*

(A) Depth profile of known low light adapted strain '*Prochlorococcus sp. MIT 0801*' (NCBITaxon:1501269) used as the response variable in an elastic net linear regression analysis searching for low light adapted *Cyanobacteria* strains from HOT Aloha 224-283 samples. The X-axis shows the relative abundance of the count values for the strain. (B) Depth profile of '*Prochlorococcus marinus str. NATL1A*' (NCBITaxon:167555), discovered in an elastic net linear regression analysis searching for low light adapted *Cyanobacteria* strains from HOT Aloha 224-283 samples. (C) Depth profile of '*Prochlorococcus marinus str. NATL2A*' (NCBITaxon:59920), discovered in an elastic net linear regression analysis searching for low light adapted *Cyanobacteria* strains from HOT Aloha 224-283 samples. (D) Depth profile of '*Prochlorococcus marinus str. MIT 9211*' (NCBITaxon:93059), discovered in an elastic net linear regression analysis searching for low light adapted *Cyanobacteria* strains from HOT Aloha 224-283 samples. (E) Depth profile of '*Prochlorococcus marinus subsp. marinus str. CCMP1375*' (NCBITaxon:167539), discovered in an elastic net linear regression analysis searching for low light-adapted *Cyanobacteria* strains from HOT Aloha 224-283 samples.

Figure 4: Top Species From OMZ-Affiliated Phyla Increasing with Decreasing Oxygen

Top 25 species resulting from multiple elastic net linear regression analyses, regressing multiple OMZ affiliated Phyla against measured '*concentration of dioxygen in liquid water*' (ENVO:3100011) values. The X-axis shows the absolute values of negative Z-scaled species coefficients derived from the regression analyses. Negative coefficients from the regression analyses represent trends of increasing abundance with decreasing oxygen concentrations. Samples used in the analyses were from the '*marine mesopelagic zone*' (ENVO:00000213) between depths of 300-600 meters from the Tara Oceans dataset.

Figure 5: System overview figure. (A) The original Planet Microbe database, available from [27], served as the source of data used in this work. (B) The functional and taxonomic analysis GitHub repository, available from [28] was used to compute the functional and taxonomic annotations from the input metagenomic datasets. The final function and taxonomic annotation outputs computed with this pipeline, deposited to the Zenodo data repository are available from [40]. (C) The system leverages subsets of ontologies drawn from the OBO Foundry Library, which are available from [136], as well as the Planet Microbe Ontology, available from [42]. (D) The Semantic Web analysis GitHub repository, available from [44], contains scripts to generate the RDF database back-end for the novel web service API, as well as query and analyze data subsets retrieved from the system. Finally, the protocol detailing how to use the system is available from [26].

Figure 6: Members of Select Phyla Most Different in Marine and River Samples '*Alphaproteobacteria*' (NCBITaxon:28211), '*Archaea*' (NCBITaxon:2157), and '*Verrucomicrobia*' (NCBITaxon:74201) species found to be most differentially abundant between the river and marine samples binned by '*Dissolved Inorganic Carbon*' (PMO:00000142) values, as determined by elastic net linear regression analyses. Samples are drawn from multiple datasets, including Amazon Plume Metagenomes, Amazon River Metagenomes, HOT 224-283, and BATS Chisholm. The X-axis shows normalized, and Aitchison transformed species counts.

Figure 7: Members of Select Gene Families Most Different in Marine and River Samples Genes from '*biosynthetic process*' (GO:0009058), '*carbohydrate catabolic process*' (GO:0016052), '*carbohydrate derivative metabolic process*' (GO:1901135), and '*transmembrane transport*' (GO:0055085) families were found to be most differentially abundant between the river and marine samples binned by '*Dissolved Inorganic Carbon*' (PMO:00000142) values, as determined by elastic net linear regression analyses. Samples are drawn from multiple datasets,

including Amazon Plume Metagenomes, Amazon River Metagenomes, HOT 224-283, and BATS Chisholm. The X-axis shows normalized, and Aitchison transformed gene counts.

Supplementary Figure 1: Identification of sequencing depth cut-offs values. Y-axes show GO and NCBITaxon annotation richness (total number of annotations). The X-axis shows the total number of reads analyzed.

Supplementary Figure 2: Identification of low-quality samples. The Y-axis shows the taxonomic annotation richness in counts based on NCBITaxon annotations. The X-axis indicates the number of ORFs.

Supplementary Figure 3: HOT Aloha 224-283 Oxygen Concentration Depth Profile. The X-axis shows measured '*concentration of dioxygen in liquid water*' (ENVO:3100011) values measured in micromoles per kilogram. The Y-axis shows water column depth in meters.

Supplementary Figure 4: Known Functional and Taxonomic Annotations. (A) Box plot showing percentage of known functional genomic (InterPro) annotations across datasets. (B) Box plot showing percentage of known taxonomic (NCBITaxon) annotations across datasets.

Table 1: Genes indicating anoxic conditions

**GO family**	**ID**	**label**	**Association Statistic**	**P value**	**Significance Code**
binding	GO:0031072	heat shock protein binding	0.87	0.001	***

binding	GO:0003690	double-stranded DNA binding	0.771	0.05	*
cellular metabolic process	GO:0006547	histidine metabolic process	1	0.001	***
cellular metabolic process	GO:0051479	mannosylglycerate biosynthetic process	0.988	0.001	***
cellular metabolic process	GO:0009061	anaerobic respiration	0.979	0.001	***
cellular metabolic process	GO:0019605	butyrate metabolic process	0.96	0.001	***
cellular metabolic process	GO:0030908	protein splicing	0.768	0.043	*
oxidoreductase activity	GO:0018492	carbon-monoxide dehydrogenase (acceptor) activity	1	0.001	***

oxidoreductase activity	GO:0042279	nitrite reductase (cytochrome, ammonia-forming) activity	0.971	0.001	***
oxidoreductase activity	GO:0016730	oxidoreductase activity, acting on iron-sulfur proteins as donors	0.929	0.001	***
oxidoreductase activity	GO:0018662	phenol 2-monooxygenase activity	0.916	0.003	**
oxidoreductase activity	GO:0030058	amine dehydrogenase activity	0.742	0.035	*

Results of indicator species analysis, following the methods of Cáceres and Legendre, for three gene families, ‘*binding*’ (GO:0005488), ‘*cellular metabolic process*’ (GO:0044237), and ‘*oxidoreductase activity*’ (GO:0016491) from anoxic ‘*marine mesopelagic zone*’ (ENVO:00000213) samples collected between 300 to 600-meter depths from the Tara Oceans dataset.

Click here to access/download;Table;Table1.xlsx

GO family	ID
binding	GO:0031072
binding	GO:0003690
cellular metabolic process	GO:0006547
cellular metabolic process	GO:0051479
cellular metabolic process	GO:0009061
cellular metabolic process	GO:0019605
cellular metabolic process	GO:0030908
oxidoreductase activity	GO:0018492
oxidoreductase activity	GO:0042279
oxidoreductase activity	GO:0016730
oxidoreductase activity	GO:0018662
oxidoreductase activity	GO:0030058

label	stat	p.value
heat shock protein binding	0.87	0.001
double-stranded DNA binding	0.771	0.05
histidine metabolic process	1	0.001
mannosylglycerate biosynthetic process	0.988	0.001
anaerobic respiration	0.979	0.001
butyrate metabolic process	0.96	0.001
protein splicing	0.768	0.043
carbon-monoxide dehydrogenase (acceptor) activity	1	0.001
nitrite reductase (cytochrome, ammonia-forming) activity	0.971	0.001
oxidoreductase activity, acting on iron-sulfur proteins as donors	0.929	0.001
phenol 2-monooxygenase activity	0.916	0.003
amine dehydrogenase activity	0.742	0.035

sig.code
***
*
***
***
***
***
*
***
***
***
**
*

Figure 1

Click here to access/download;Figure;Figure1.png

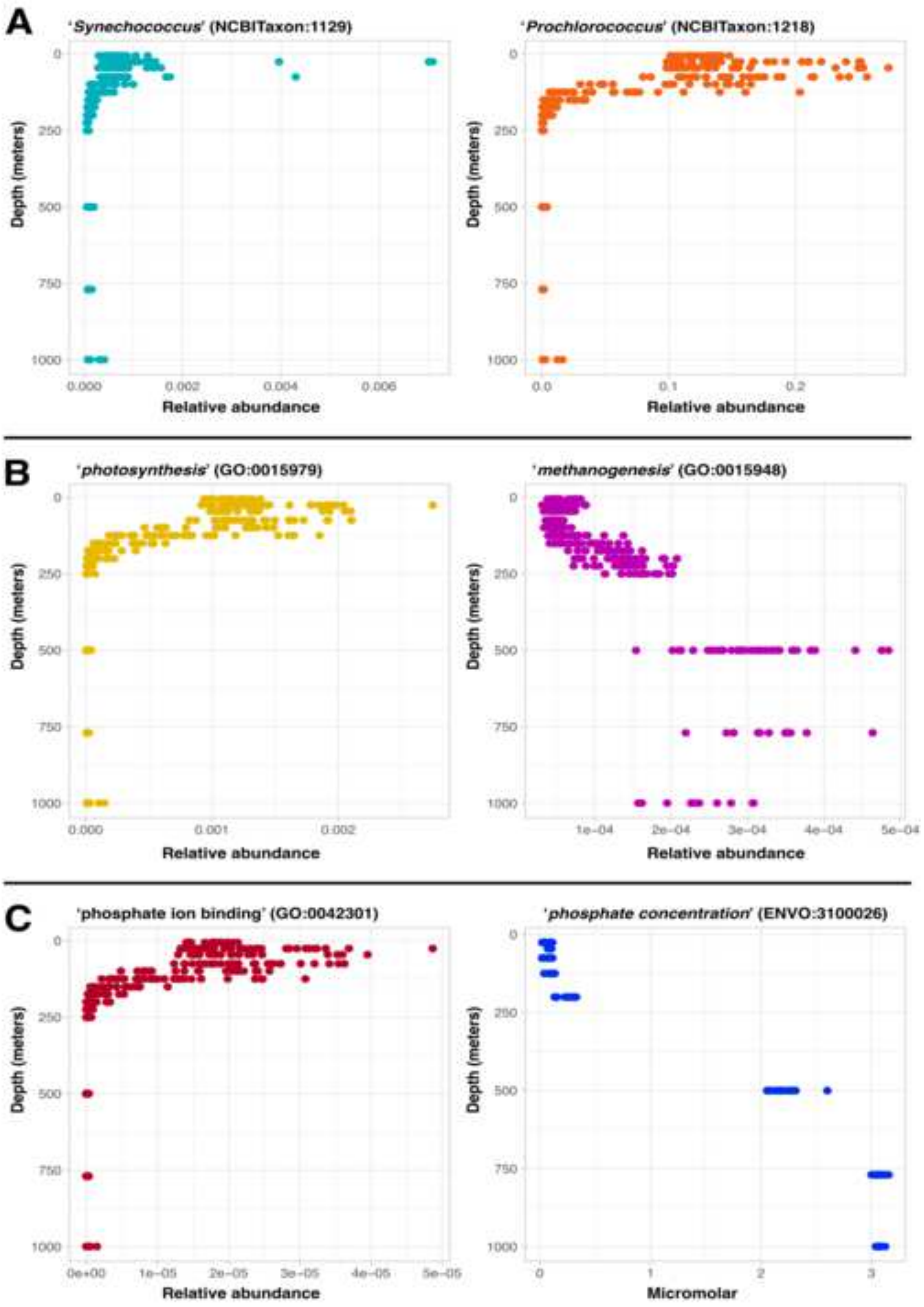

Figure 2

Click here to access/download;Figure;Figure2.png

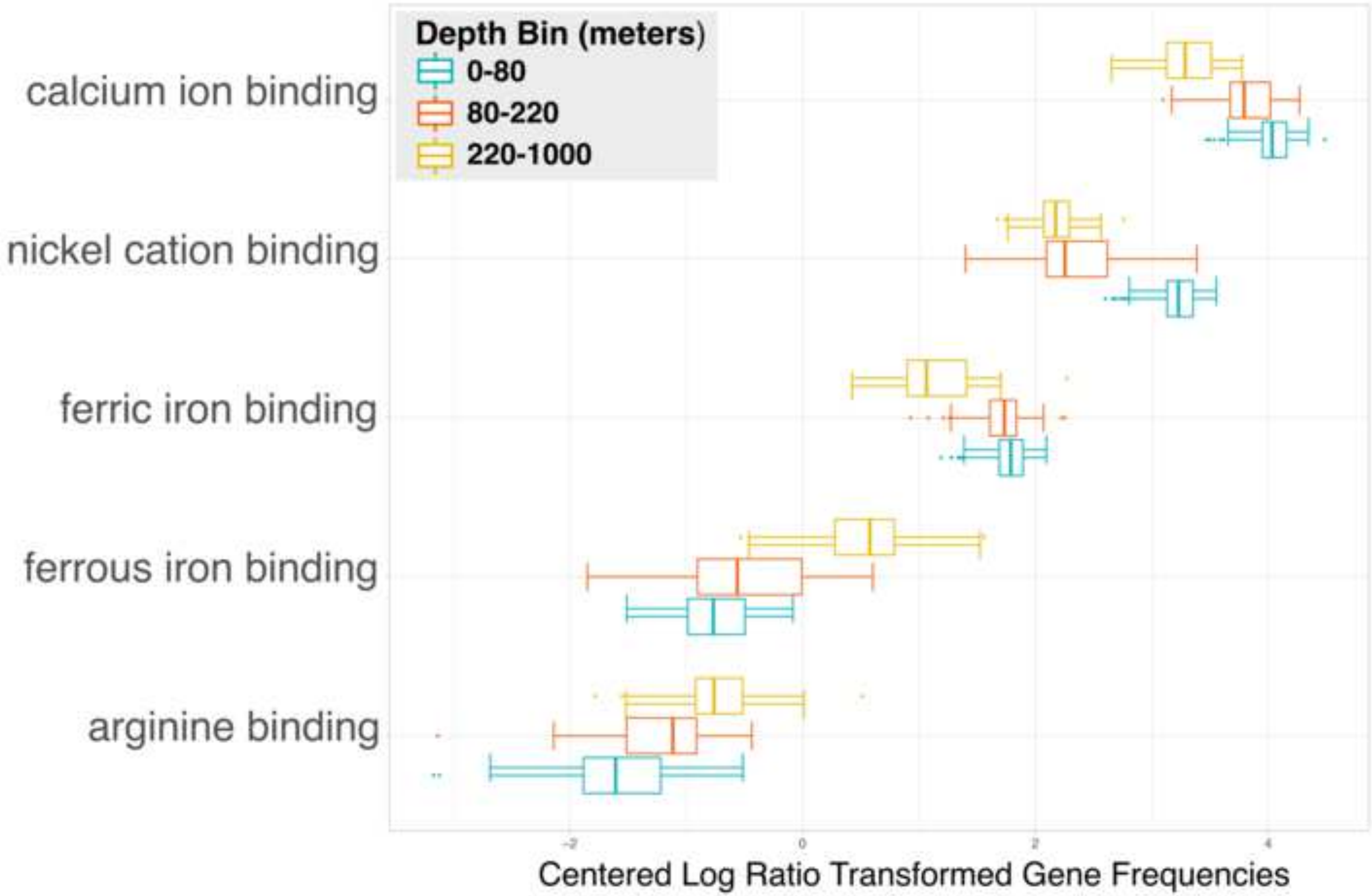

Figure 3

Click here to access/download;Figure;Figure3.png

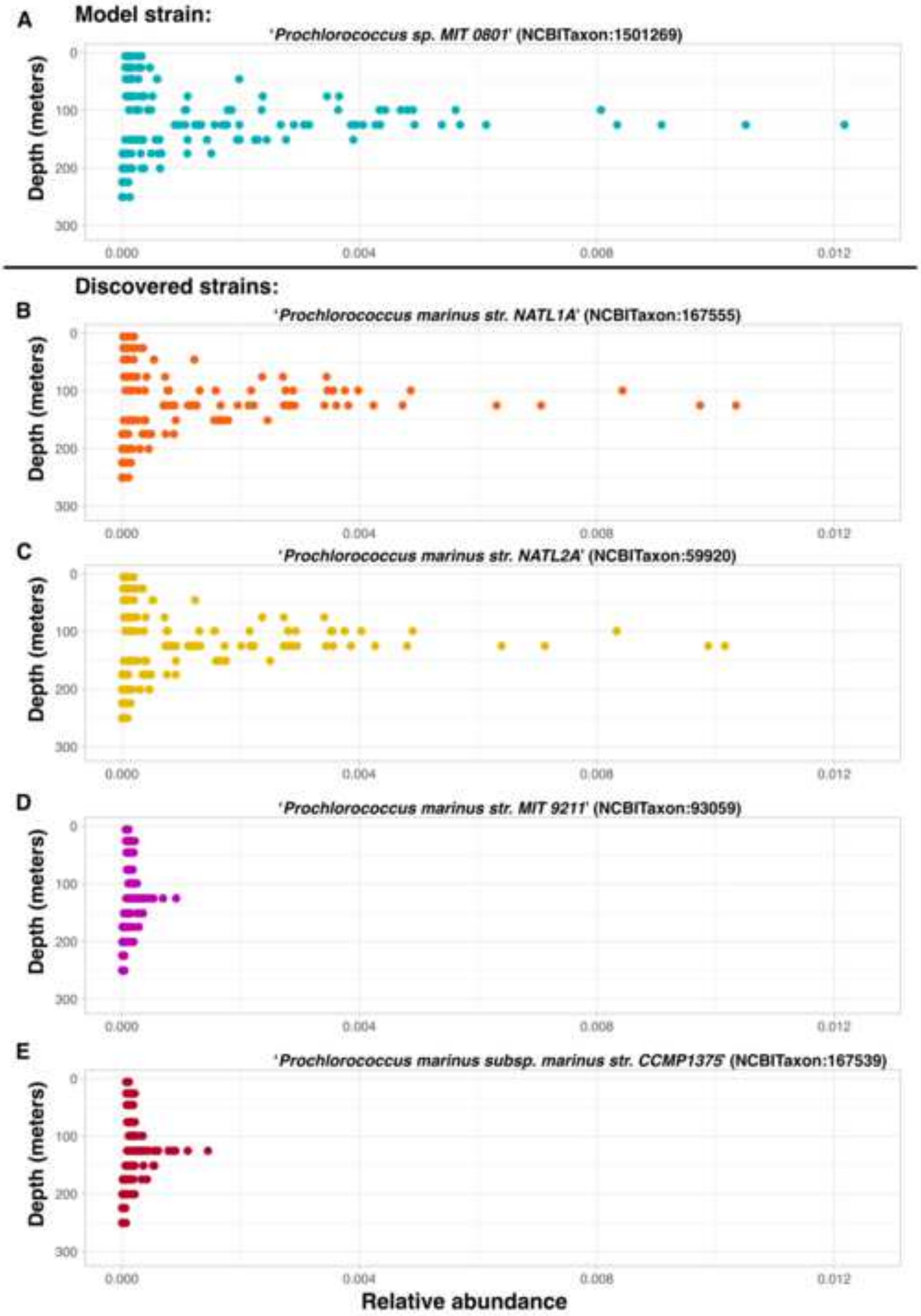

Figure 4

Click here to access/download;Figure;Figure4.png

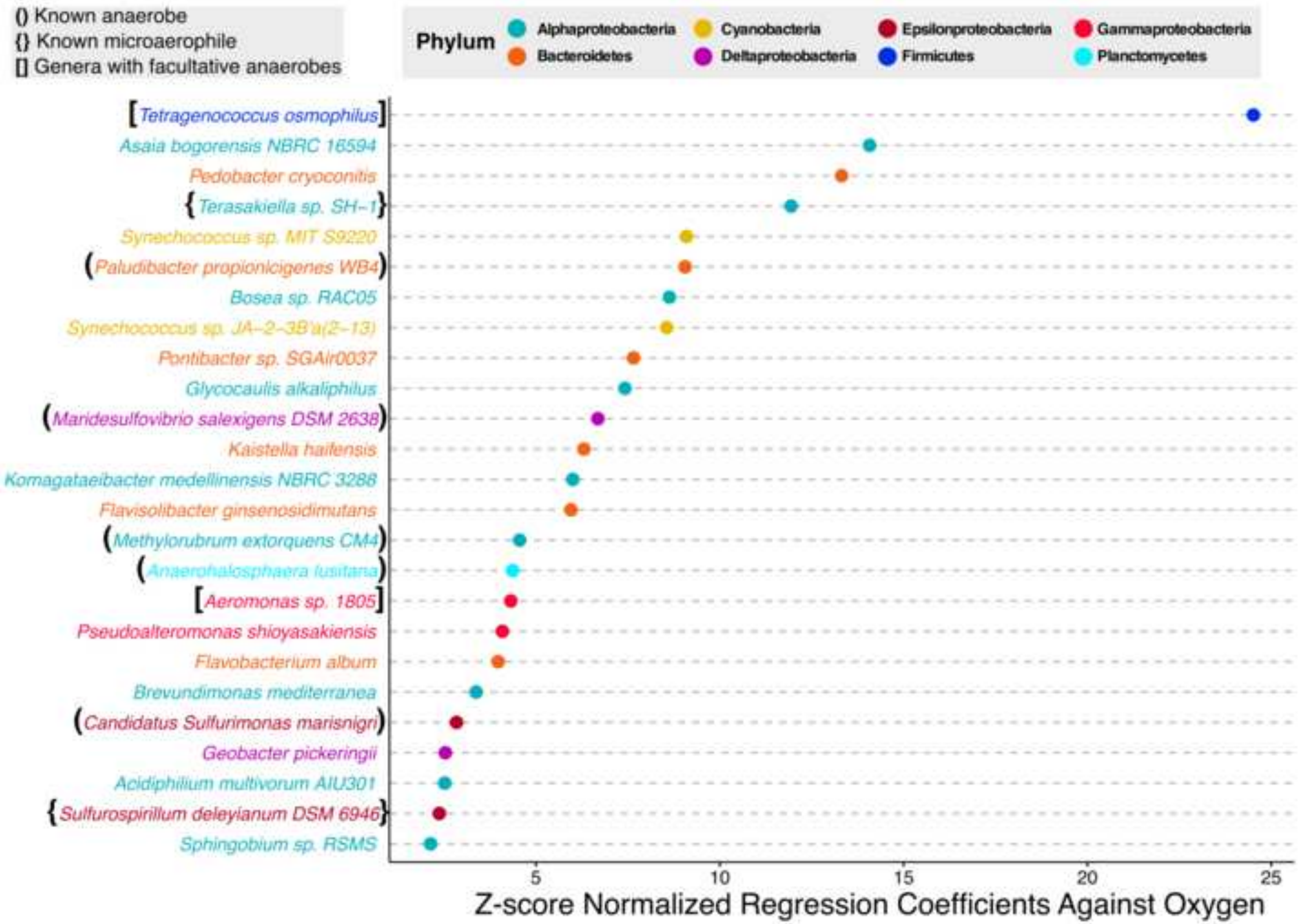

Figure 5

Click here to access/download;Figure;Figure5.png

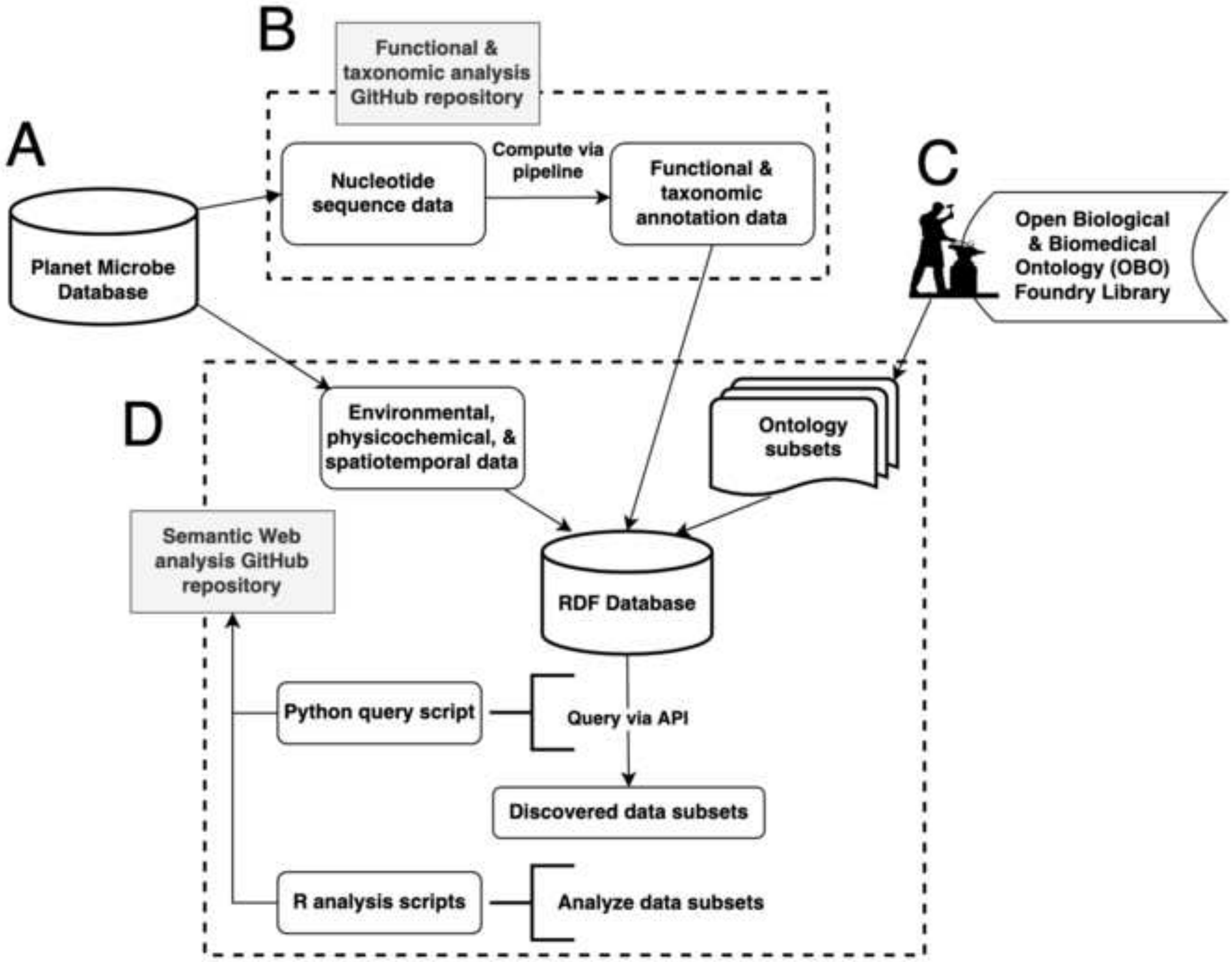

Figure 6

Click here to access/download;Figure;Figure6.png

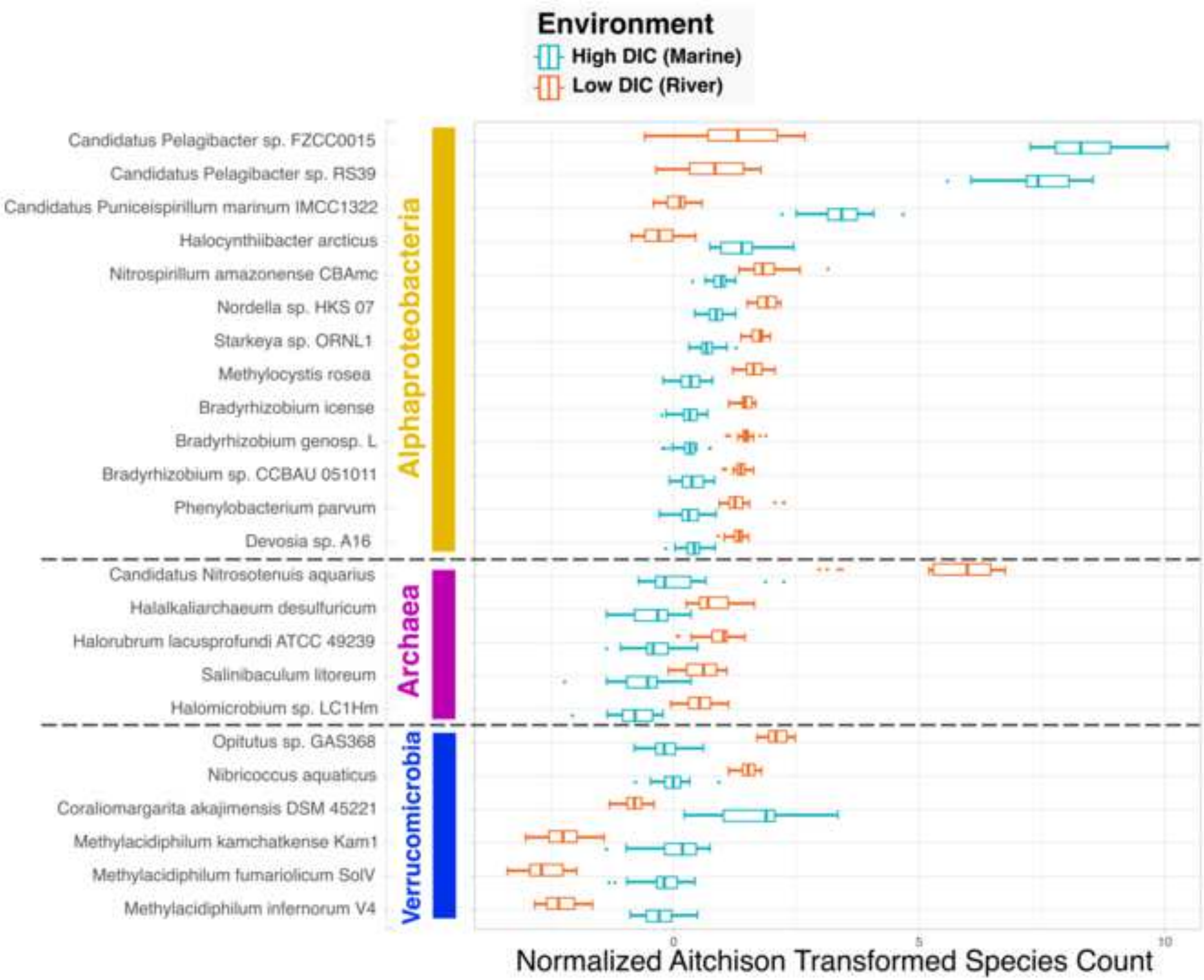

Figure 7

Click here to access/download;Figure;Figure7.png

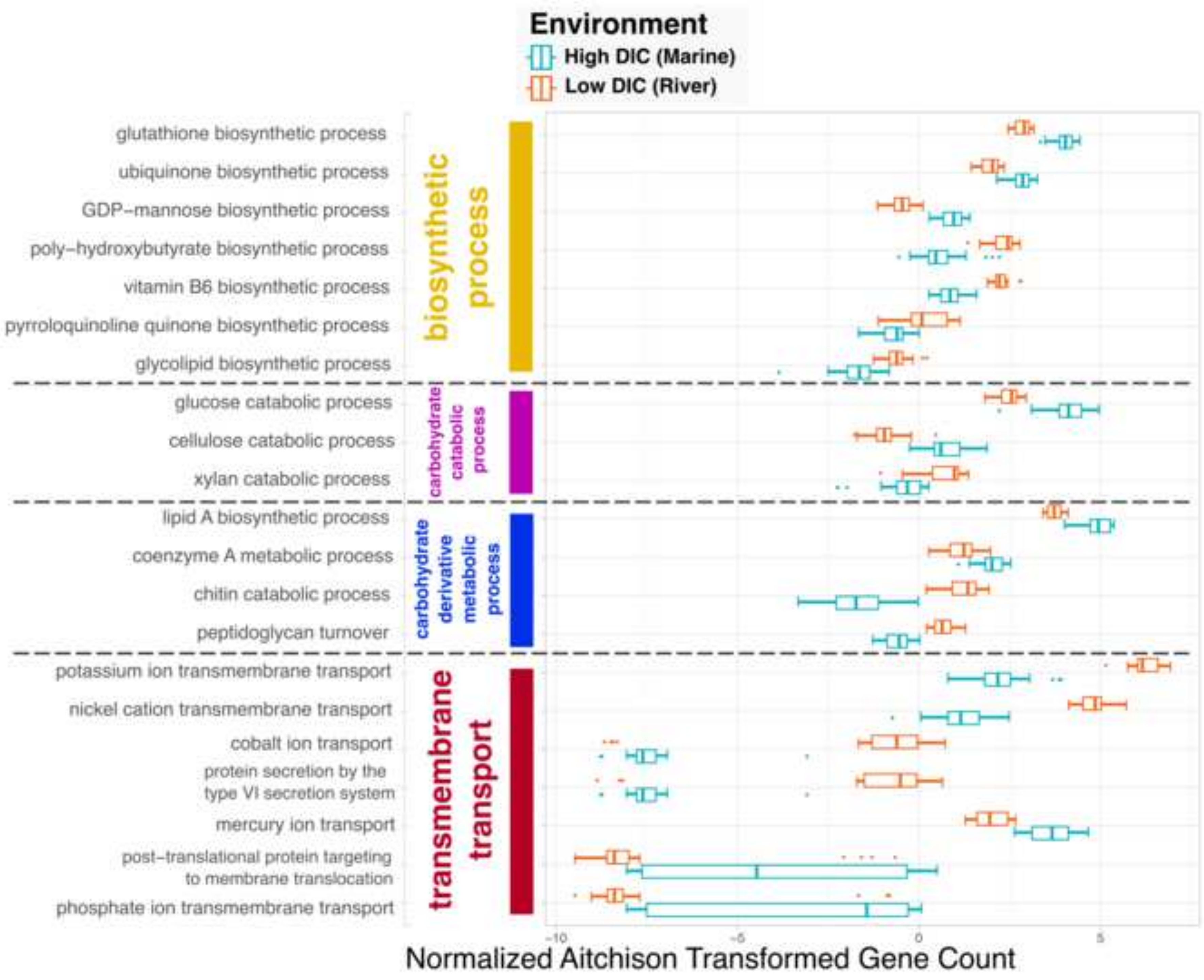

Supplementary Material 1

Click here to access/download

Supplementary Material

Supplementary_Material_Figure_1.png

Supplementary Material 2

Click here to access/download
Supplementary Material
Supplementary_Material_Figure_2.png

Click here to access/download
Supplementary Material
Supplementary_Material_Figure_3.png

Supplementary Material 4

Click here to access/download
Supplementary Material
Supplementary_Material_Figure_4.png
