# Supplementary figures and images for: Ontology-driven analysis of marine metagenomics: what more can we learn from our data?
Source: Gigascience. 2023 Nov 6;12:giad088. doi: 10.1093/gigascience/giad088 (PMC10632069; doi:10.1093/gigascience/giad088)

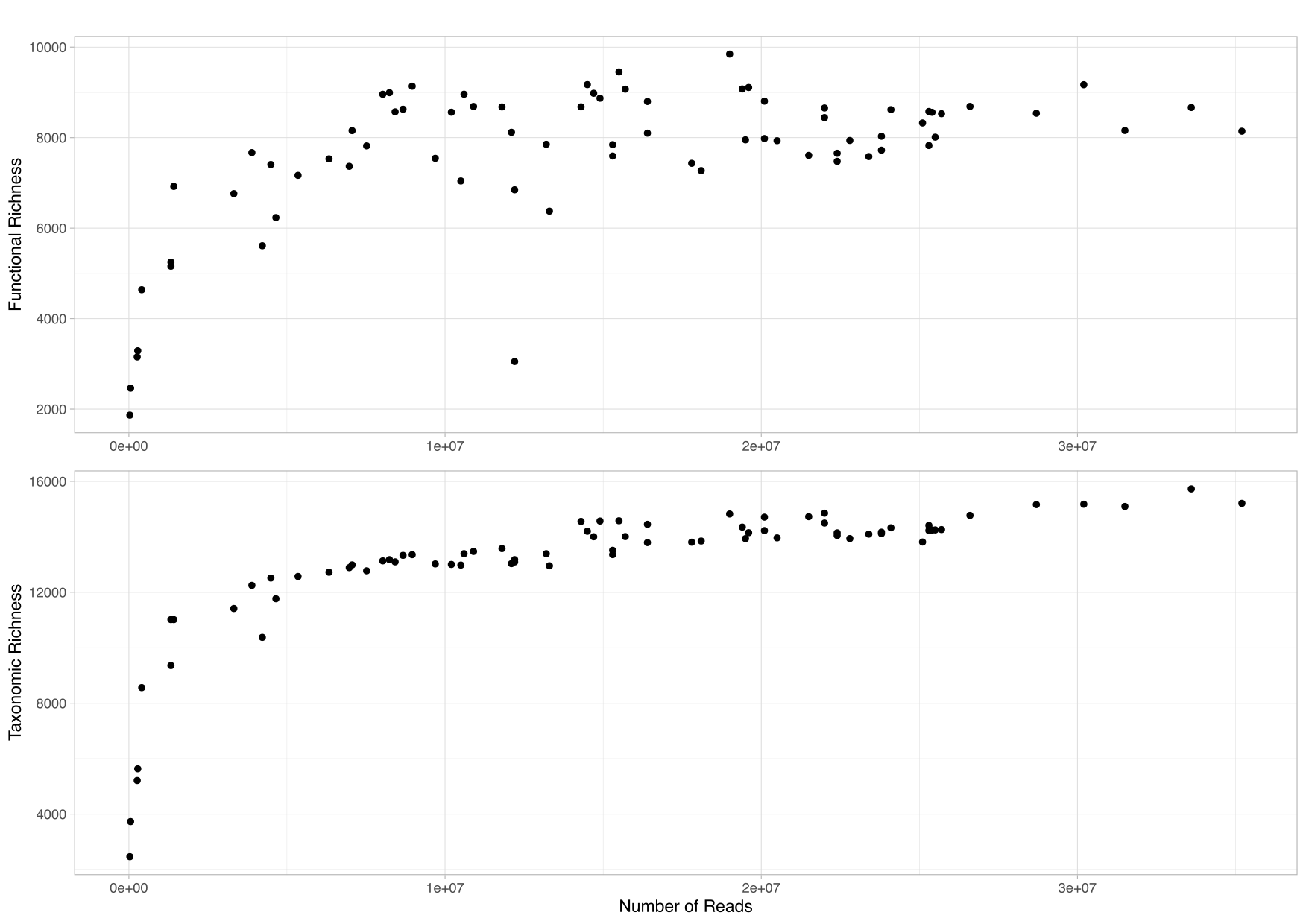

Supplement: giad088_Supplemental_Figures [file giad088_supplemental_figures.zip › Supplementary_Material_Figure_1.png]

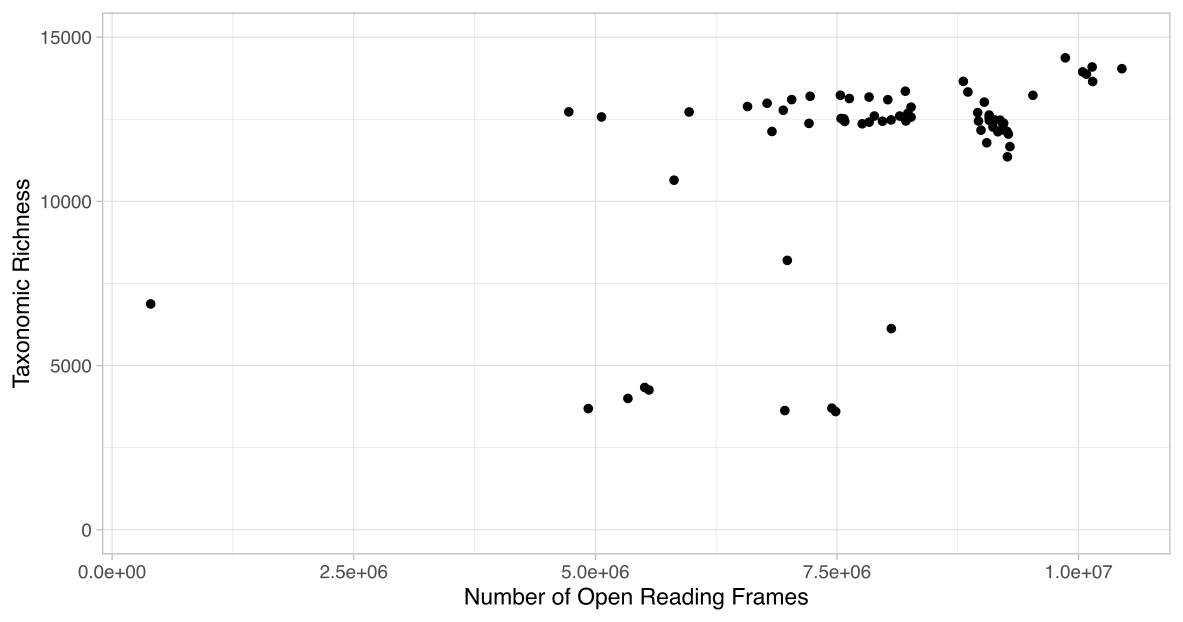

Supplement: giad088_Supplemental_Figures [file giad088_supplemental_figures.zip › Supplementary_Material_Figure_2.png]

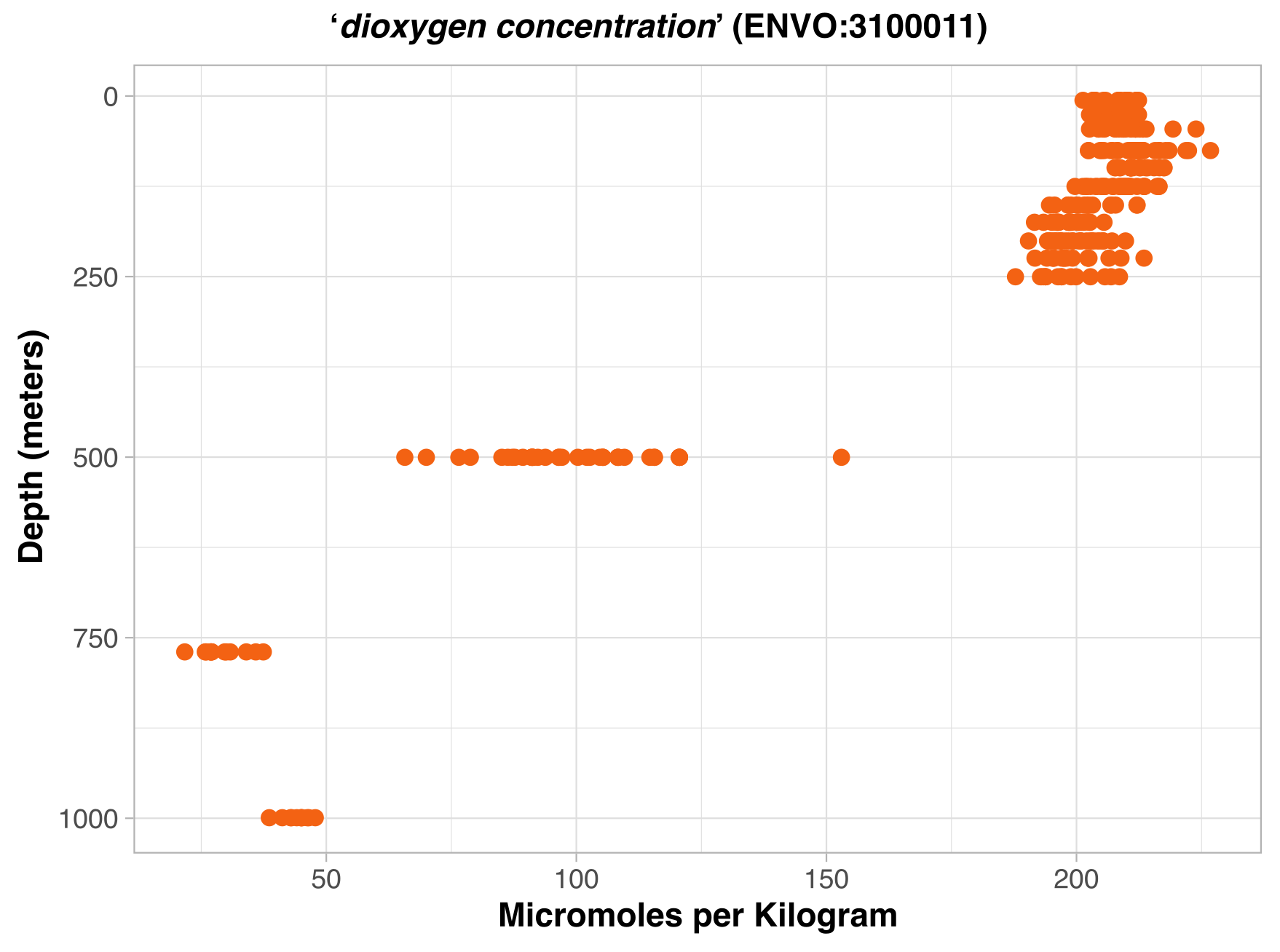

Supplement: giad088_Supplemental_Figures [file giad088_supplemental_figures.zip › Supplementary_Material_Figure_3.png]

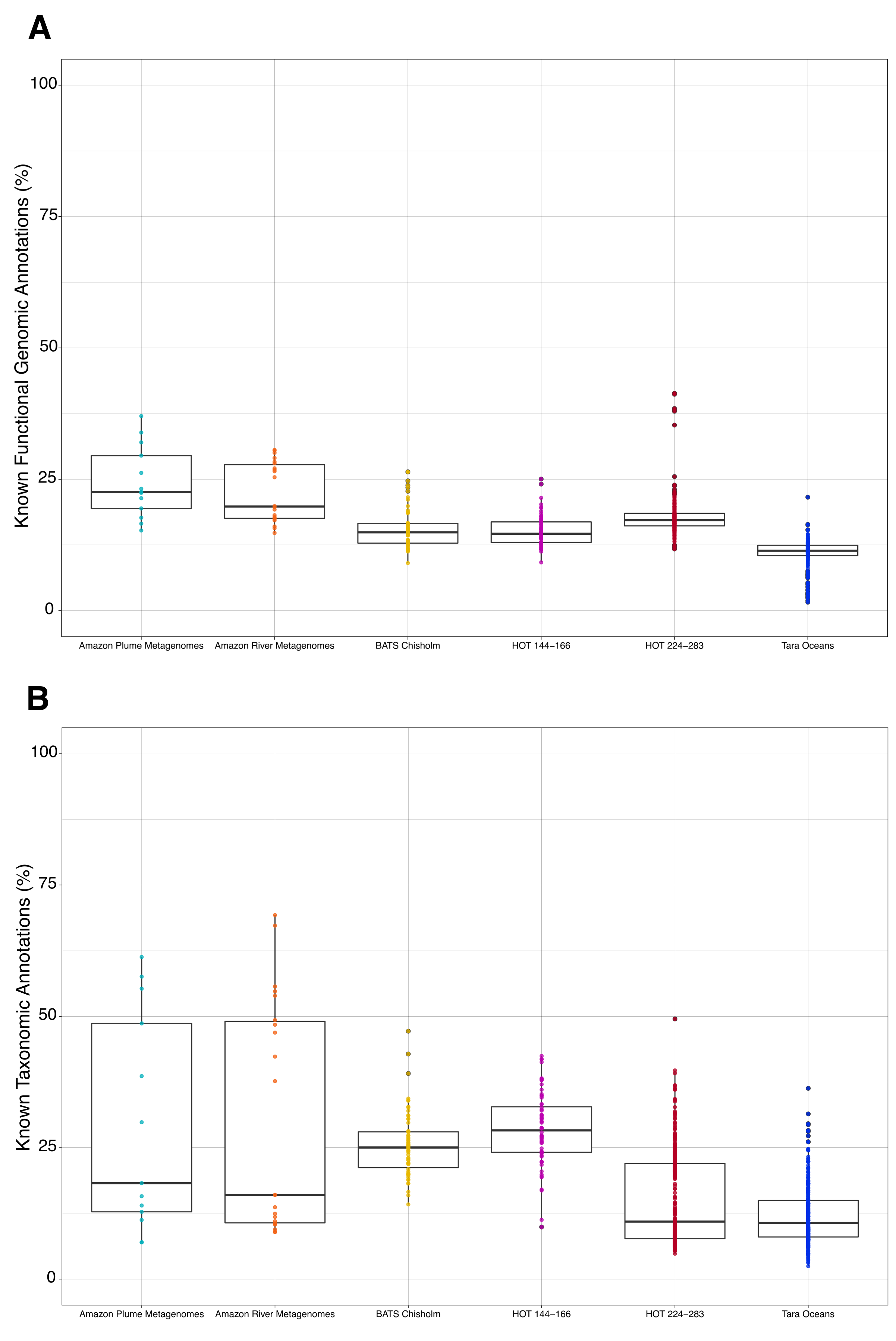

Supplement: giad088_Supplemental_Figures [file giad088_supplemental_figures.zip › Supplementary_Material_Figure_4.png]
