# Supplementary material for: Ontology-driven analysis of marine metagenomics: what more can we learn from our data?
Source: Gigascience. 2023 Nov 6;12:giad088. doi: 10.1093/gigascience/giad088 (PMC10632069; doi:10.1093/gigascience/giad088)
Supplement: giad088_Response_to_Reviewer_Comments_Original_Submission [file giad088_response_to_reviewer_comments_original_submission.pdf]

Author's Response To Reviewer Comments

Reviewer #1 Response:

We thank the reviewer for their interest and for their time in reviewing this manuscript. We appreciate their kind comments and conclusions.

Reviewer #2 Response:

> With the proliferation of microbiome data, it has become important to have databases and services to organize and more important make data discoverable. In this manuscript, the authors developed a service integrating metagenomic datasets with ontologies and showcased its application for asking novel biological questions.
The methods presented in the manuscript seem appropriate to the aim of the work and globally well described (despite few concerns raised below). The results they obtained are really interesting and their conclusion supported by the data. The possible future applications look promising.

We thank the reviewer for their time and interest in reviewing this manuscript. We hope that this work can help provide some guidelines for future applications that leverage ontology-based workflows for the analysis of large scale metagenomic datasets.

> The manuscript is easy to read but I have some reservations about its clarity. It is listed as a research paper but the main output of this work seems to be "a novel web service accessible through an open API that can be used to ask novel biological questions of existing metagenomic datasets.", even if the result section offers a lot of interesting results but that are more applications of the developed service.

We thank the reviewer for this important comment. Indeed, we agree that we could better clarify the main output of this work. Our intention with this manuscript is to demonstrate the potential for this type of ontology-driven Semantic Web based metagenomic application. In order to put forward the outcome that we think is the most important, we rephrased the section as follows:
"To fill this gap, we built upon the previously published Planet Microbe web portal to propose a novel FAIR architecture for the ontology-driven harmonization and meta-analysis of large-scale marine metagenomic datasets. We demonstrate how this type of effort can enable the discovery and retrieval of data about the environmental context, functional genomic potential, and taxonomic structure of the marine prokaryotic microbiome through an API service."

We also reflected this change in the abstract where we've changed the text to be: "Most importantly, we present and evaluate a novel Semantic Web architecture that can be used to ask novel biological questions of existing metagenomic datasets."

> If the main output is really the service. It could be improved and easier to use. It is not clear why this service is not directly integrated in Planet Microbe. This could give it a proper interface that would also help users to use it.

We thank the reviewer for this comment. As stated in our previous response, the main output is not the web service. Regarding integration with the original Planet Microbe database, we decided to keep the two databases separate for multiple reasons: firstly, Planet Microbe contains datasets that are not included in this new architecture (in particular 16S rRNA amplicon datasets, or WGS datasets below the determined sequencing depth). Additionally, the two databases have very different structures and usage, making the integration of both systems difficult. The original Planet Microbe database links environmental physicochemical and spatiotemporal information to metagenomic samples, whereas the new database links a subset of those original metadata features to functional and taxonomic annotations computed on a subset of the original metagenomic data. The original database uses a non-SQL relational database architecture, whereas the current work uses an RDF and Semantic Web technology-based architecture. Although the new database was generated using data from the first and both use a common semantic layer

(with OBO ontologies), the two database's architectures are incompatible (Postgress and RDF). Thus, the new RDF database cannot be directly integrated within the original Planet Microbe web portal without completely changing the original web portal.

To the point of ensuring continuity between original Planet Microbe database web portal and the new work conducted in this manuscript, we have added the following text box to the original Planet Microbe website describing this new project building upon original Planet Microbe database: "Ontology-Driven Analysis of Marine Metagenomics: What More Can We Learn from Our Data? In the latest phase of Planet Microbe research suite, we integrate functional and taxonomic annotations computed on the prokaryotic fraction of the Planet Microbe database with life-science ontologies into a novel FAIR Semantic Web database. The new system leverages the intersection of ontologies and the computed data to ask novel biological questions such as "What data do we have about metagenomes from the 'HOT 224-283' project, where we have observed occurrences of 'cellular lipid metabolic process'(es) [GO:0044255], as well as recorded 'water temperature' [ENVO:09200014] values?". Check it out for yourself following the protocol we created [here](http://dx.doi.org/10.17504/protocols.io.e6nvwkw19vmk/v2)." We additionally created a documentation section for this work on the original Planet Microbe gitbook documentation page.

Finally, regarding the creation of a Graphical User Interface (GUI) for the new work, unfortunately this is not something that can easily be done or would necessarily work well for a number of reasons. As described in the protocol.io protocol, queries to the RDF system require the use of a python script which includes various command line arguments to assemble a complex query. Important arguments for the script include the use of ontology terms from various open-source ontologies. Although there exist websites to view and browse the ontologies, (as described in the protocol), integrating those types of ontology browser tools directly into a single GUI would be very challenging and is outside the scope of this work. Additionally, not all system queries happen instantaneously, so accommodations for longer system queries would have to be built into the interface which would involve creating an interface for job submission queues and job completion emails etc. Unfortunately, without developer support this would be a considerable workload. We think the reviewer is absolutely right to point out that our main output is not a fully flushed service with a user interface, but rather the creation of a new database architecture that allows for ontology driven analyses of microbiome data. Thus, the manuscript's primary output is the creation and validation of the new system, as well as demonstrating its ability to retrieve data leading to new biological results.

> An overview of the work and its connection with Planet Microbiome would be also nice to have as a figure.

We thank the reviewer for this great suggestion. We agree that the connection between this work and Planet Microbe was unclear in the original submission. Thus, we have added a figure to better show the interaction between this new resource and Planet Microbe. The new overview figure (new Figure 5) describes the global workflow of the system, and its caption summarizes all hyperlinks to the relevant repositories, databases, and protocol. We think the new figure 5 helps visualize the global steps of this work and should hopefully address the reviewers' concerns about the connection between the current work and the original Planet Microbe database, as well clarify what was done in this work.

> I also found the documentation really limited. The GitHub repository would benefit from some reorganization, adding the requirements (maybe using package management system), usage description and contribution guidelines, and maybe packaging the scripts.

We thank the reviewer for raising this important point. We have updated the github repository documentation pages associated with this manuscript as recommended. We added sections for Description, Requirements, Installation, Contributions, Usage to both github repositories. Regarding package management systems, the functional and taxonomic annotation pipeline makes use of a conda environment for version control of the pipeline tools, as well as snakemake workflow to facilitate running the pipeline. We have clarified this in the GitHub's readme file. Finally, we have also made versioned releases of both github repositories.

Note that the main piece of documentation for this work is the protocols.io page

http://dx.doi.org/10.17504/protocols.io.e6nvwkw19vmk/v2 describing how to use/reuse the novel FAIR architecture to retrieve and analyze data as described in the results of this manuscript. Although that protocol was originally linked in the Semantic Web analysis GitHub repository's readme file and methods section of the manuscript, we agree that it wasn't completely clear that the protocols.io page is the main piece of documentation associated with this work. As mentioned in the previous response this is now better clarified in the caption of the new overview figure. The protocol is also now more clearly mentioned in the last paragraph of the introduction, in addition to its mention in the Data Availability section. We hope this clarifies any confusion about the works documentation. We put considerable effort into making the protocols.io documentation page. We hope that this protocol makes this work accessible for others to reproduce the results of the work, as well as perform new system queries if desired.

> It should be clarified if it is possible and how to expand with new datasets, or how to update when new ontologies and reference databases are available.

We thank the reviewers for raising these important questions. Unfortunately, at the moment it is not possible to add new ontologies or data to the current version of system, without first computing the new data through the functional and taxonomic annotation pipeline, updating the ontology subsets, and producing a new version of the RDF database. We hope to be able to make new releases of the RDF database in the future with more data as well as updated ontology subsets. To clarify these points in the manuscript we have added these details to the future directions section.

"Regarding future directions for this work, as new datasets are made available in Planet Microbe database, their functional and taxonomic annotations can be computed through the pipeline described in this manuscript. This data, along with new releases of the ontologies can be used to generate future releases of the RDF database. This would enable the systems to be used to ask and answer novel biological questions over an expanded range of ecosystems and physicochemical gradients. Additionally, the FAIR architecture for the ontology-driven harmonization and meta-analysis metagenomic datasets presented in this work could be applied in novel studies to scientific domains beyond marine and aquatic environments, enabling novel insights into microbiome of terrestrial, engineered, or host-associated environments."

> L. 65: The term management is probably better than stewardship.

Thank you for your suggestion, the 2016 Wilkinson et al FAIR paper uses both words stewardship and management, so we were reusing their language, but we think the reviewer is right to prefer management rather than stewardship. We have updated the manuscript accordingly.

> It would be great to have the citation of the FAIR principles before

Agreed. We have added the following sentence: "Regarding data management there has been much discussion of the FAIR principles (Findability, Accessibility, Interoperability, and Reusability), which outline general principles for improving the digital ecosystem supporting the publication of scientific data." as citation seven (moved up from being citation 13) and modified the original line 64 to reflect this change.

> L. 113: The database created is only accessible via an API, but no link is provided

We thank the reviewer for this comment. Instead of just putting the link to the API, which requires instructions provided in the protocol, we added the link to the documentation in the introduction line 114. The use of the API is documented in the protocols.io link in the Data Availability section. That protocol describes how to query the API using custom python scripts (provided in the https://github.com/hurwitzlab/planet-microbe-semantic-web-analysis github repository). This is further clarified in the newly added overview figure describing the global workflow of the manuscript. The caption of this new figure provides links to all resources used in the manuscript including the link to the protocols.io page detailing how to use the system.

> L 132: A justification for the subsampling of the selected metagenomes is missing

We thank the reviewer for raising this point. The richness curves shown in Supplementary Figure 1 shows

that the ratio of new functional and taxonomic annotations seen with increased sequencing depth starts to plateau after 10 million reads. We have updated the text around Line 132 accordingly to explain this.

> L 155: Bracken is mentioned in the README file but not in the manuscript

We thank the reviewer for raising this point. We included both Bracken and Kraken within the computational pipeline. Both programs are computationally rapid, k-mer based taxonomic identification tools, so we included both of them in the computational pipeline. Bracken does a taxonomic reassignment of Kraken results from lower taxonomic levels of resolution (e.g., order or class) to assignments at species-level based on probability distributions within the data. However, as our ontology-based RDF search architecture is able to discover results at any level of taxonomic assignment, we chose to use the more conservative Kraken taxonomic results rather than the probabilistically re-assigned Bracken taxonomic results in the database.

> L. 179: What are the subsets of GO and NCBITaxon ontologies? On which criteria?

We thank the reviewer for asking us to clarify this point. We created subsets of the GO and NCBITaxon ontologies that only included the functional and taxonomic terms obtained from the analysis as well as their parent terms within the ontology. Doing this reduced the search space of ontology terms for which we did not have data, increasing the querying speed of the RDF system. We have clarified this in the manuscript text in the sentence before the original line 179.

> L. 189: How has the RDF graph been modified?

We thank the reviewer for raising the question. The answer is quite specific to the RDF codebase, therefore, rather than detailing how the final RDF graph structure used in this work is different from the mentioned graph shown in https://github.com/jamesaoverton/cob-data-demo/blob/master/doc/proposal-1.md, we chose to include the link to the appendix of the planet-microbe-semantic-web-application protocol’s Appendix 2, which shows examples of the final RDF structure used in this work, and not mention the original graph. We have modified the text as follows (after the original line 189).

“The SPARQL construct queries used to build the RDF graph database can be found in the “triplestore” subdirectory of the https://github.com/hurwitzlab/planet-microbe-semantic-web-analysis/ github repository. Examples illustrating the RDF graph structure used in this work can be found in the Appendix 2 of the protocol shown in the Data Availability section. “

> L. 196: A link to the Planet Microbe database API would be nice to have there

We thank the reviewer for their suggestion, we’ve added a link to the API to that line.

> L. 211: "By the number of ORFs for genes" is misleading

We thank the reviewer for their feedback, we agree. By this we meant dividing by the number of ORFs to normalize the functional annotation count values. We clarified this by adding some new text to the sentence preceding the original line 211.

> L. 211: "Number of sample reads"?

We thank the reviewer for their question, by this we mean the number of reads each sample had. We have clarified this in the new text added after the original line 211.

> L. 211: What is the quality control for species?

We thank the reviewer again for their comment, here we meant the number of reads that remained after the taxonomic pipeline’s quality control steps which include running bowtie2 Trimmomatic and vsearch, as described in the “Functional and Taxonomic Metagenomic Annotation” section of the methods. We have changed the sentence after the original line 211 to the following to clarify:

“The functional and taxonomic results shown in all figures except for Table 1, were normalized as follows. Functional annotation count values were normalized by dividing each sample’s GO count values by the number of ORFs annotated by the pipeline for each sample. Taxonomic annotation count values were normalized by dividing each sample’s NCBITaxon count values by the number of reads that remained after the taxonomic pipeline’s quality control steps for each sample.”

> L. 214: The sentence sounds like a repetition of the one before (same in L.221)

We thank the reviewer for catching this typo. The duplications were removed.

> L. 227: It might be good to add "centered log-ratio" between Aitchison and transformation

We thank the reviewer for their suggestion, we have included centered log-ratio in parentheses between Aitchison and transformation in the sentence on the original line 227.

> L. 238: An explanation for the choice Elastic Net Linear Regression analyses would be great to have

We thank the reviewer for their suggestion. We have added an explanation for our choice of the Elastic net method for doing linear regression to a sentence on the original line 240. In short, Elastic net combines features from both lasso and ridge regression methods, each of which are popular methods for linear regression.

> L. 240: A justification for using a 10-fold cross-validation method could be added

We thank the reviewer for their comments. The glmnet r package that we used for linear regression uses 10-fold cross validation as the default for the partitioning of data when selecting model coefficients for the final regression analysis. Hence, we simply used the software with default parameters as suggested by the authors in their documentation https://glmnet.stanford.edu/articles/glmnet.html. We have updated the text on the original line 240 to be the following:

“We performed the linear regression analyses using the default glmnet parameters including the use of 10-fold cross-validation, as well as the selection of a regularization parameter lambda such that the cross-validated error is within one standard error of the minimum in order to determine the model coefficients.”

> L. 243: The recommendation should be cited

Thank you for catching this, we have added the citation to the sentence on the original line 243.

> L. 775: Wrong licences for code

We thank the reviewer for catching this, we originally had Creative Commons 4.0 licenses on both repositories. We then modified the license to be The MIT License (MIT) as per request of the data audit editor, however, we subsequently didn’t update the manuscript. Thank you for noticing this. We have updated the manuscript accordingly to reflect the correct licenses on both github repositories.

> Missing tool/database version …

We thank the reviewer for catching these omissions, we have added version numbers for the following tools as you mentioned Kraken2 Prodigal and Pfam, as well as well as the Composition, ggplot2, cor, adonis/vegan, glmnet and indicspecies R packages.

> Functional and Taxonomic Metagenomic Annotation: GitHub repository
The README file could be more clear. It currently mentions only the functional annotation
It could be versioned, with authors and contributors mentioned

We thank the reviewer for their feedback, you're absolutely right that it wasn’t clear that the functional

annotation github repository was doing both functional and taxonomic annotation. We have updated the repository accordingly to clarify this. As previously mentioned in our response, we added sections for Description, Requirements, Installation, Contributions, Usage to both github repositories, as well as made versioned releases of both github repositories. For the planet-microbe-functional-annotation repository, we created an install script that handles installing all the required software and conda environments. Additionally, we have included directions for editing and running the pipeline, descriptions of the various parameters, and cleaned up the code and readme. Finally, we have added a tutorial with a small file illustrating the steps needed to run the pipeline.

> For reproducibility, a package management system like conda could be used by providing an environment for all tools, not just some

We thank the reviewer for these important suggestions, as stated in a previous response, the functional and taxonomic annotation pipeline uses a conda environment for version control of the pipeline tools, as well as snakemake workflow by which to run the pipeline, which is now made clear in the readme file of the relevant GitHub repository.

We have added FragGeneScan, vsearch, and Trimmomatic to the conda environment. However, we elected to keep the installation of InterProScan separate so that users can utilize the most up-to-date version of InterProScan, when reusing this pipeline for new applications. As of writing this, InterProScan is at v5.62-94.0, while the conda version of InterProScan is v5.59-91.0. Our install.sh script downloads the latest version of InterProScan for the user, it is noted in the manuscript that we used version 5.46-81.0.

> Scripts could be provided instead of code in README file

We thank the reviewer for this suggestion. We have created a script `install.sh` for users to run to install everything needed for the pipeline.

> What about using a workflow management system as recommended in the Author guidelines? And having them in protocols.io?

We thank the reviewer for their comment. As mentioned in our response above, the pipeline is indeed using a snakemake workflow management system. We made this more apparent in the readme of the repository. Thanks to the reviewer comments we have improved the README file for the functional/taxonomic annotation pipeline, including more detailed information about reusing a snakemake pipeline.
