# Supplementary material for: Ontology-driven analysis of marine metagenomics: what more can we learn from our data?
Source: Gigascience. 2023 Nov 6;12:giad088. doi: 10.1093/gigascience/giad088 (PMC10632069; doi:10.1093/gigascience/giad088)
Supplement: giad088_Response_to_Reviewer_Comments_Revision_1 [file giad088_response_to_reviewer_comments_revision_1.pdf]

Author's Response To Reviewer Comments

We have updated the manuscript with the few minor formatting and editorial requests.
