# Supplementary material for: Ontology-driven analysis of marine metagenomics: what more can we learn from our data?
Source: Gigascience. 2023 Nov 6;12:giad088. doi: 10.1093/gigascience/giad088 (PMC10632069; doi:10.1093/gigascience/giad088)
Supplement: giad088_Reviewer_1_Report_Original_Submission — Neil Davies, Ph.D -- 4/3/2023 Reviewed [file giad088_reviewer_1_report_original_submission.pdf]

Reviewer Report

Title: Ontology-Driven Analysis of Marine Metagenomics: What More Can We Learn from Our Data?

Version: Original Submission **Date:** 4/3/2023

Reviewer name: Neil Davies, Ph.D.

Reviewer Comments to Author:

The authors highlight the importance of making metagenomic data discoverable within machine searchable frameworks. They propose a novel web service that integrates marine metagenomic datasets with community-driven life-science ontologies. This service addresses the need to maximize the knowledge that society is gaining from the proliferation of metagenomic sequencing of the marine environment. It presents advances in cyberinfrastructure based on the FAIR data principles and importantly the use of ontologies as powerful ways of presenting knowledge in ways that facilitate reuse through machines. Critical to this is the support of open APIs to the cyberinfrastructure. The authors intend to promote the capacity for researchers to test hypotheses about the structure and function of microbial communities in the oceans and the paper makes a compelling case that the new service advances this goal.

The paper demonstrates the importance of having state-of-the-art data sets from well-studied locations. Using the Hawaiian Ocean Time Series data and its relatively well understood ecosystem, for example, they put the system through its paces and demonstrate its considerable utility in enabling complex search and analysis combining taxonomic, ecological, environmental and chemical parameters to explore ecosystem function in meaningful ways (e.g., methanogenesis is one process they investigate and one could imagine the importance of such knowledge for society facing the challenge of greenhouse gas emissions).

In conclusion, the paper makes a valuable contribution and in my opinion could be published without revisions.

Methods

Are the methods appropriate to the aims of the study, are they well described, and are necessary controls included? Choose an item.

Conclusions

Are the conclusions adequately supported by the data shown? Choose an item.

Reporting Standards

Does the manuscript adhere to the journal's guidelines on minimum standards of reporting? Choose an item.

Choose an item.

Statistics

Are you able to assess all statistics in the manuscript, including the appropriateness of statistical tests used? Choose an item.

Quality of Written English

Please indicate the quality of language in the manuscript: Choose an item.

Declaration of Competing Interests

Please complete a declaration of competing interests, considering the following questions:

- Have you in the past five years received reimbursements, fees, funding, or salary from an organisation that may in any way gain or lose financially from the publication of this manuscript, either now or in the future?
- Do you hold any stocks or shares in an organisation that may in any way gain or lose financially from the publication of this manuscript, either now or in the future?
- Do you hold or are you currently applying for any patents relating to the content of the manuscript?
- Have you received reimbursements, fees, funding, or salary from an organization that holds or has applied for patents relating to the content of the manuscript?
- Do you have any other financial competing interests?
- Do you have any non-financial competing interests in relation to this paper?

If you can answer no to all of the above, write 'I declare that I have no competing interests' below. If your reply is yes to any, please give details below.

I declare that I have no competing interests

I agree to the open peer review policy of the journal. I understand that my name will be included on my report to the authors and, if the manuscript is accepted for publication, my named report including any attachments I upload will be posted on the website along with the authors' responses. I agree for my report to be made available under an Open Access Creative Commons CC-BY license (http://creativecommons.org/licenses/by/4.0/). I understand that any comments which I do not wish to be included in my named report can be included as confidential comments to the editors, which will not be published.

Choose an item.

To further support our reviewers, we have joined with Publons, where you can gain additional credit to further highlight your hard work (see: https://publons.com/journal/530/gigascience). On publication of this paper, your review will be automatically added to Publons, you can then choose whether or not to claim your Publons credit. I understand this statement.

Yes Choose an item.
