# Supplementary material for: Ontology-driven analysis of marine metagenomics: what more can we learn from our data?
Source: Gigascience. 2023 Nov 6;12:giad088. doi: 10.1093/gigascience/giad088 (PMC10632069; doi:10.1093/gigascience/giad088)
Supplement: giad088_Reviewer_2_Report_Original_Submission — BÃ©rÃ©nice Batut, Ph.D. -- 4/26/2023 Reviewed [file giad088_reviewer_2_report_original_submission.pdf]

Reviewer Report

Title: Ontology-Driven Analysis of Marine Metagenomics: What More Can We Learn from Our Data?

Version: Original Submission **Date:** 4/26/2023

Reviewer name: BÃ©rÃ©nice Batut, Ph.D.

Reviewer Comments to Author:

With the proliferation of microbiome data, it has become important to have databases and services to organize and more important make data discoverable. In this manuscript, the authors developed a service integrating metagenomic datasets with ontologies and showcased its application for asking novel biological questions.
The methods presented in the manuscript seem appropriate to the aim of the work and globally well described (despite few concerns raised below). The results they obtained are really interesting and their conclusion supported by the data. The possible future applications look promising.
The manuscript is easy to read but I have some reservations about its clarity. It is listed as a research paper but the main output of this work seems to be "a novel web service accessible through an open API that can be used to ask novel biological questions of existing metagenomic datasets.", even if the result section offers a lot of interesting results but that are more applications of the developed service.
If the main output is really the service. It could be improved and easier to use. It is not clear why this service is not directly integrated in Planet Microbe. This could give it a proper interface that would also help users to use it. An overview of the work and its connection with Planet Microbiome would be also nice to have as a figure.
I also found the documentation really limited. The GitHub repository would benefit from some reorganisation, adding the requirements (maybe using package management system), usage description and contribution guidelines, and maybe packaging the scripts. It should be clarified if it is possible and how to expand with new datasets, or how to update when new ontologies and reference databases are available.
Detailed comments
L. 65: The term management is probably better than stewardship. It would be great to have the citation of the FAIR principles before
L. 113: The database created is only accessible via an API, but no link is provided
L 132: A justification for the subsampling of the selected metagenomes is missing
L 155: Bracken is mentioned in the README file but not in the manuscript
L. 179: What are the subsets of GO and NCBITaxon ontologies? On which criteria?
L. 189: How has the RDF graph been modified?
L. 196: A link to the Planet Microbe database API would be nice to have there
L. 211: "By the number of ORFs for genes" is misleading
L. 211: "Number of sample reads"?
L. 211: What is the quality control for species?
L. 214: The sentence sounds like a repetition of the one before (same in L.221)

L. 227: It might be good to add "centered log-ratio" between Aitchison and transformation
L. 238: An explanation for the choice Elastic Net Linear Regression analyses would be great to have
L. 240: A justification for using a 10-fold cross-validation method could be added
L. 243: The recommendation should be cited
L. 775: Wrong licences for code
Missing tool/database version
L. 152: Kraken2
L. 161: Prodigal
L. 165: Pfam
L. 228: Composition R package
L. 232: ggplot2 package
L. 233: cor and adonis packages
L. 234: vegan package
L. 239: glmnet package
L. 250: indicspecies package
Functional and Taxonomic Metagenomic Annotation: GitHub repository
The README file could be more clear. It currently mentions only the functional annotation
It could be versioned, with authors and contributors mentioned
For reproducibility, a package management system like conda could be used by providing an environment for all tools, not just some
Scripts could be provided instead of code in README file
What about using a workflow management system as recommended in the Author guidelines? And having them in protocols.io?

Methods

Are the methods appropriate to the aims of the study, are they well described, and are necessary controls included? Choose an item.

Conclusions

Are the conclusions adequately supported by the data shown? Choose an item.

Reporting Standards

Does the manuscript adhere to the journal's guidelines on [minimum standards of reporting?](#) Choose an item.

Choose an item.

Statistics

Are you able to assess all statistics in the manuscript, including the appropriateness of statistical tests used? Choose an item.

Quality of Written English

Please indicate the quality of language in the manuscript: Choose an item.

Declaration of Competing Interests

Please complete a declaration of competing interests, considering the following questions:

- Have you in the past five years received reimbursements, fees, funding, or salary from an organisation that may in any way gain or lose financially from the publication of this manuscript, either now or in the future?
- Do you hold any stocks or shares in an organisation that may in any way gain or lose financially from the publication of this manuscript, either now or in the future?
- Do you hold or are you currently applying for any patents relating to the content of the manuscript?
- Have you received reimbursements, fees, funding, or salary from an organization that holds or has applied for patents relating to the content of the manuscript?
- Do you have any other financial competing interests?
- Do you have any non-financial competing interests in relation to this paper?

If you can answer no to all of the above, write 'I declare that I have no competing interests' below. If your reply is yes to any, please give details below.

I declare that I have no competing interests

I agree to the open peer review policy of the journal. I understand that my name will be included on my report to the authors and, if the manuscript is accepted for publication, my named report including any attachments I upload will be posted on the website along with the authors' responses. I agree for my report to be made available under an Open Access Creative Commons CC-BY license (http://creativecommons.org/licenses/by/4.0/). I understand that any comments which I do not wish to be included in my named report can be included as confidential comments to the editors, which will not be published.

Choose an item.

To further support our reviewers, we have joined with Publons, where you can gain additional credit to further highlight your hard work (see: https://publons.com/journal/530/gigascience). On publication of this paper, your review will be automatically added to Publons, you can then choose whether or not to claim your Publons credit. I understand this statement.

Yes Choose an item.
